# Comprehensive definition of human immunodominant CD8 antigens in tuberculosis

**DOI:** 10.1038/s41541-017-0008-6

**Published:** 2017-04-03

**Authors:** Deborah A. Lewinsohn, Gwendolyn M. Swarbrick, Byung Park, Meghan E. Cansler, Megan D. Null, Katelynne G. Toren, Joy Baseke, Sarah Zalwango, Harriet Mayanja-Kizza, LaShaunda L. Malone, Melissa Nyendak, Guanming Wu, Kristi Guinn, Shannon McWeeney, Tomi Mori, Keith A. Chervenak, David R. Sherman, W. Henry Boom, David M. Lewinsohn

**Affiliations:** 1 Division of Infectious Diseases, Department of Pediatrics, Oregon Health & Science University, Portland, OR USA; 2 Knight Cancer Institute, Oregon Health & Science University, Portland, OR USA; 3 Uganda-CWRU Research Collaboration, Kampala, Uganda; 4 Department of Medicine, Makerere University College of Health Sciences, Kampala, Uganda; 5 Tuberculosis Research Unit, Case Western Reserve University, Cleveland, OH USA; 6 Division of Infectious Diseases, Department of Medicine, Oregon Health & Science University, Portland, OR USA; 7 Center for Infectious Disease Research, formerly Seattle Biomedical Research Institute, Seattle, WA USA; 8 Department of Global Health, University of Washington, Seattle, WA USA; 9 Division of Pulmonary and Critical Care Medicine, Oregon Health & Science University, Portland, OR USA; 10 Portland Veterans Administration Medical Center, Portland, OR USA; 11 Present Address: University of Washington, Seattle, WA USA; 12 Kampala City Council, Kampala, Uganda; 13 Guinn consulting, Denver, CO USA

## Abstract

Despite widespread use of the Bacillus Calmette-Guerin vaccine, tuberculosis, caused by infection with *Mycobacterium tuberculosis*, remains a leading cause of morbidity and mortality worldwide. As CD8^+^ T cells are critical to tuberculosis host defense and a phase 2b vaccine trial of modified vaccinia Ankara expressing Ag85a that failed to demonstrate efficacy, also failed to induce a CD8^+^ T cell response, an effective tuberculosis vaccine may need to induce CD8^+^ T cells. However, little is known about CD8, as compared to CD4, antigens in tuberculosis. Herein, we report the results of the first ever HLA allele independent genome-wide CD8 antigen discovery program. Using CD8^+^ T cells derived from humans with latent tuberculosis infection or tuberculosis and an interferon-γ ELISPOT assay, we screened a synthetic peptide library representing 10% of the *Mycobacterium tuberculosis* proteome, selected to be enriched for *Mycobacterium tuberculosis* antigens. We defined a set of immunodominant CD8 antigens including part or all of 74 *Mycobacterium tuberculosis* proteins, only 16 of which are previously known CD8 antigens. Immunogenicity was associated with the degree of expression of mRNA and protein. Immunodominant antigens were enriched in cell wall proteins with preferential recognition of Esx protein family members, and within proteins comprising the *Mycobacterium tuberculosis* secretome. A validation study of immunodominant antigens demonstrated that these antigens were strongly recognized in *Mycobacterium tuberculosis*-infected individuals from a tuberculosis endemic region in Africa. The tuberculosis vaccine field will likely benefit from this greatly increased known repertoire of CD8 immunodominant antigens and definition of properties of *Mycobacterium tuberculosis* proteins important for CD8 antigenicity.

## Tuberculosis: Defining the proteins that drive immune responses

Specific bacterial proteins have been found that drive effective immune responses to tuberculosis, with use in making more effective vaccines. Immunity to tuberculosis (TB) is facilitated by two types of white blood cell; however, most research has focused on one: the CD4^+^ T cell. Deborah A. Lewinsohn and David Lewinsohn, of the Oregon Health & Science University, USA, and collaborators lay out the essential functions of the oft-neglected CD8^+^ T cell, and undertook a broad approach to catalogue and define the bacterial proteins that activate the CD8^+^ T cell response. The team found that TB-infected humans reacted strongly to their protein library, and described several characteristics of CD8^+^ T cell ‘antigens’ (activators of immune cells) that will likely prove highly useful in the design of more protective TB vaccines.

## Introduction

Infection with *Mycobacterium tuberculosis* (Mtb) remains a leading cause of infectious disease morbidity, and mortality^1^ and is a major opportunistic infection in individuals with HIV/AIDS.^2^ While more doses of the existing the tuberculosis (TB) vaccine BCG have been given worldwide than any other vaccine, 8.6 million cases and 1.3 million deaths occur annually, highlighting the need for a more effective vaccine.^1^

TB immunity relies heavily on components of the cellular immune system.^3^ Moreover, it has long been appreciated that CD4^+^ T cell immunity is critical in host defense to TB.^4^ Yet, a randomized, placebo-controlled phase 2b trial of modified vaccinia Ankara (MVA) virus expressing antigen 85 A used as a BCG booster in infants demonstrated no efficacy against TB despite the induction of modest IFN-γ, TNF-α, IL-2 producing CD4^+^ T cell responses.^5^ Of note, while MVA characteristically induces robust CD8^+^ T cell responses, this TB vaccine did not induce CD8^+^ T cell responses.

CD8^+^ T cells are associated with strong CD4^+^ TH1 cell responses, and ample experimental evidence in the mouse TB model has suggested a protective role for CD8^+^ T cells in the host response to infection.^6–8^ Mtb-specific CD8^+^ T cells have been identified in humans with TB as well as individuals with latent TB infection (LTBI).^6^ These CD8^+^ T cells are either classically (MHC-Ia) or non-classically (MHC-Ib) restricted by CD1,^9^ HLA-E,^10^ or MR1.^11^ While CD8^+^ and CD4^+^ T cells share some effector functions, such as cytolysis and IFN-γ and TNF-α secretion, CD8^+^ T cells also display unique recognition of MHC Class II negative cells, preferential recognition of heavily infected cells,^12^ the capability to induce apoptosis of infected cells,^13^ secrete granulysin (a component of the cytotoxic granule can directly inhibit Mtb growth),^14^ and control of chronic infection.^15, 16^ Thus, induction of CD8^+^ T cells may represent a valuable attribute of an effective TB vaccine.

While definition of CD8 antigens is essential for development of a TB vaccine targeting induction of CD8^+^ T cells, relatively little is known about CD8, as compared to CD4, antigens in TB. Investigation of CD8 antigens has focused on known CD4 antigens^17^ or other Mtb proteins of interest, such as proteins expressed by the Dormancy Survival Regulon (*DosR*) associated with dormancy.^18^ It is perhaps not surprising that CD8 epitopes have been reported from only 270 proteins representing 7% of the proteome, with the majority contained within 30 proteins.^19^ Broader approaches to CD8 antigen/epitope discovery are limited to a more recent report from Tang *et al*., who utilized HLA binding prediction algorithms to evaluate HLA-A*02 binding epitope and interrogated approximately 400 proteins for CD8 epitopes. They validated 18 novel CD8 epitopes in 18 proteins.^20^ Herein, rather than using HLA binding prediction algorithms, we used a weighting schema based upon a multiple attribute decision-making:framework and available genomic and proteomic information, to select *Mycobacterium tuberculosis* (Mtb) proteins most likely to represent CD8 antigens, and then designed a synthetic peptide library comprised of 15-mers overlapping by 9 aa representing the products of approximately 10% of the Mtb genome (389 of 4011 genes). Using an IFN-γ ELISPOT assay, we screened this peptide library with ex vivo CD8^+^ T cells derived from individuals with LTBI or TB. We then used these data to identify and define the characteristics of immunodominant CD8 antigens, and validated the immunogenicity of these CD8 antigens in Mtb-infected Ugandan adults.

## Results

### Human subjects enrollment for ex vivo CD8^+^ T cell screen of peptide library

For the library screen, we enrolled Mtb-infected individuals who were racially diverse, reflecting diverse genetic backgrounds of people infected with a variety of Mtb strains, as well as those who were both asymptomatically infected with Mtb (LTBI) and those with disease due to Mtb infection (TB). Therefore, we enrolled a total of 20 Mtb-infected individuals comprised of 15 with LTBI (Caucasian *n* = 6, Asian *n* = 5, African American *n* = 4) and 5 with TB disease (any race). Details of subject enrollment are summarized in Table S1.

### Mtb-infected humans demonstrate strong and broad CD8^+^ T cell responses to Mtb proteins

**Fig. 1:**
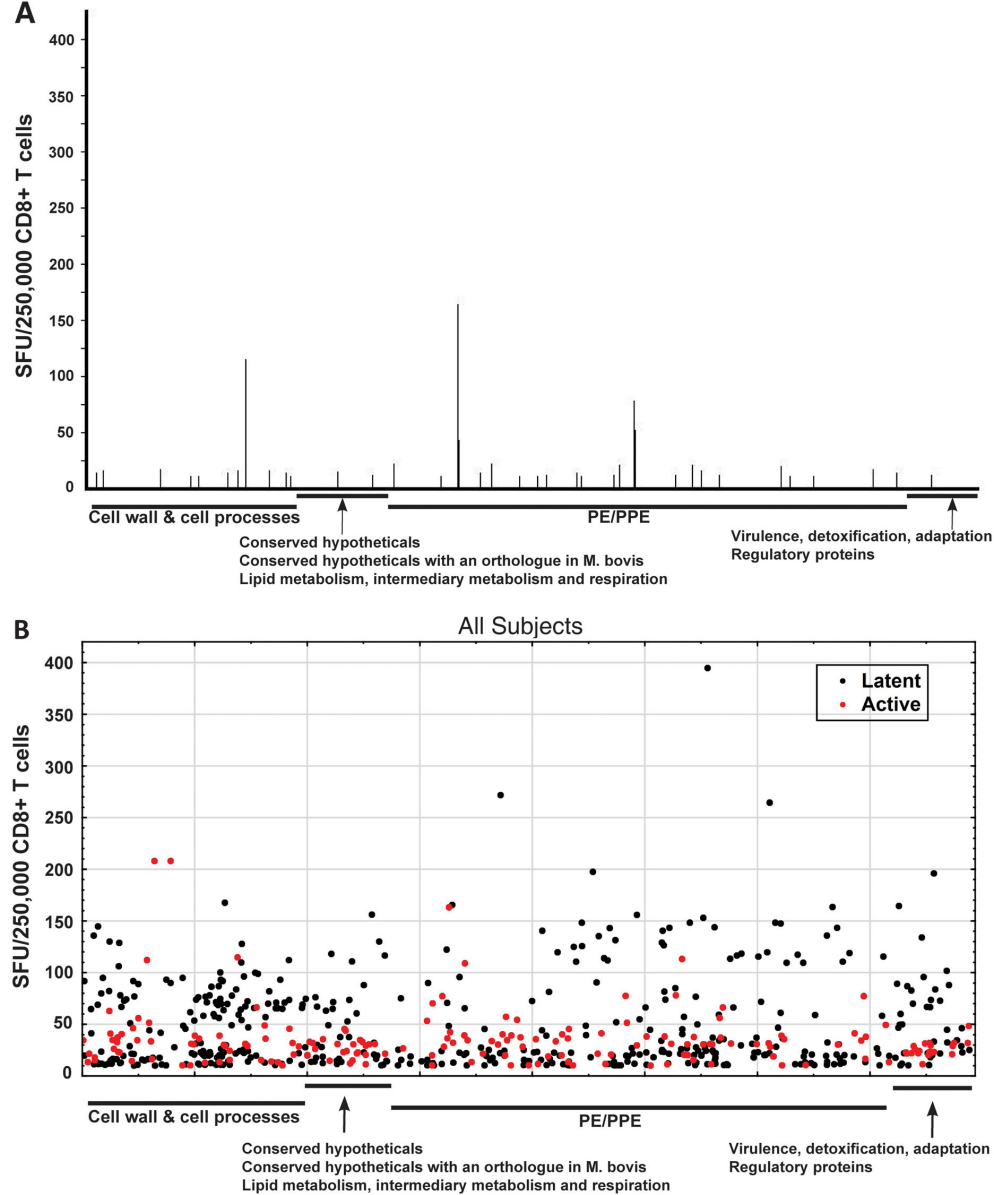
Ex vivo CD8^+^ T cell peptide library screens. **a** The SFU from positive wells (as defined in methods) from the ex vivo CD8^+^ T cell peptide library screen for each individual well are depicted. The x-axis represents each of the 789 peptide pools arrayed by Tuberculist functional classification. **b** Compiled results from ex vivo screens (*n* = 20) are depicted. The top 5% of each subject’s screen are shown. Subjects with LTBI are shown in *black* whereas individuals with TB are shown in *red*

To determine the repertoire of immunodominant CD8 antigens within the Mtb proteome, we used CD8^+^ T cells to screen ex vivo a synthetic peptide library representing approximately 10% of the Mtb proteome. As previously described,^21^ the library consisted of proteins selected for likelihood of antigenicity based upon known genomic and proteomic information. The library was comprised of 789 peptide pools representing 389 unique proteins. The number of proteins represented by an individual peptide pool ranged from 1 to 4 (Figure S1A). Conversely, as each peptide pool is comprised of 50 15-mer peptides overlapping by 9 amino acids, smaller proteins are represented by a single peptide pool (*n* = 48), while larger proteins required more than one peptide pool to represent the entire protein (Figure S1B). Eleven peptide pools represented portions of two proteins belonging to distinct Tuberculist categories. Results from a representative ex vivo screen of an individual with TB disease are shown in Fig. 1a, where each of the 789 peptide pools are denoted along the x-axis arranged by the Tuberculist functional classification. When present, ex vivo CD8^+^ T cell responses are robust. However, the array of positive responses is relatively limited within a single individual, consistent with a highly focused response. Compiled data from all twenty subjects are presented in Fig. 1b. The strongest CD8^+^ T cell responses that ranked in the top 5% of an individual’s positive responses are shown. There was no statistically significant difference between the magnitude of the response between active TB and LTBI donors (*p* = 0.2225; Mann–Whitney test). These data demonstrate great diversity in CD8^+^ T cell responses between individuals of diverse genetic backgrounds. Additionally, there is no discernable pattern noted in individuals with LTBI compared to those with TB.

### Immunodominant CD8 antigens were preferentially found in the Mtb cell wall including the esx protein family

**Fig. 2:**
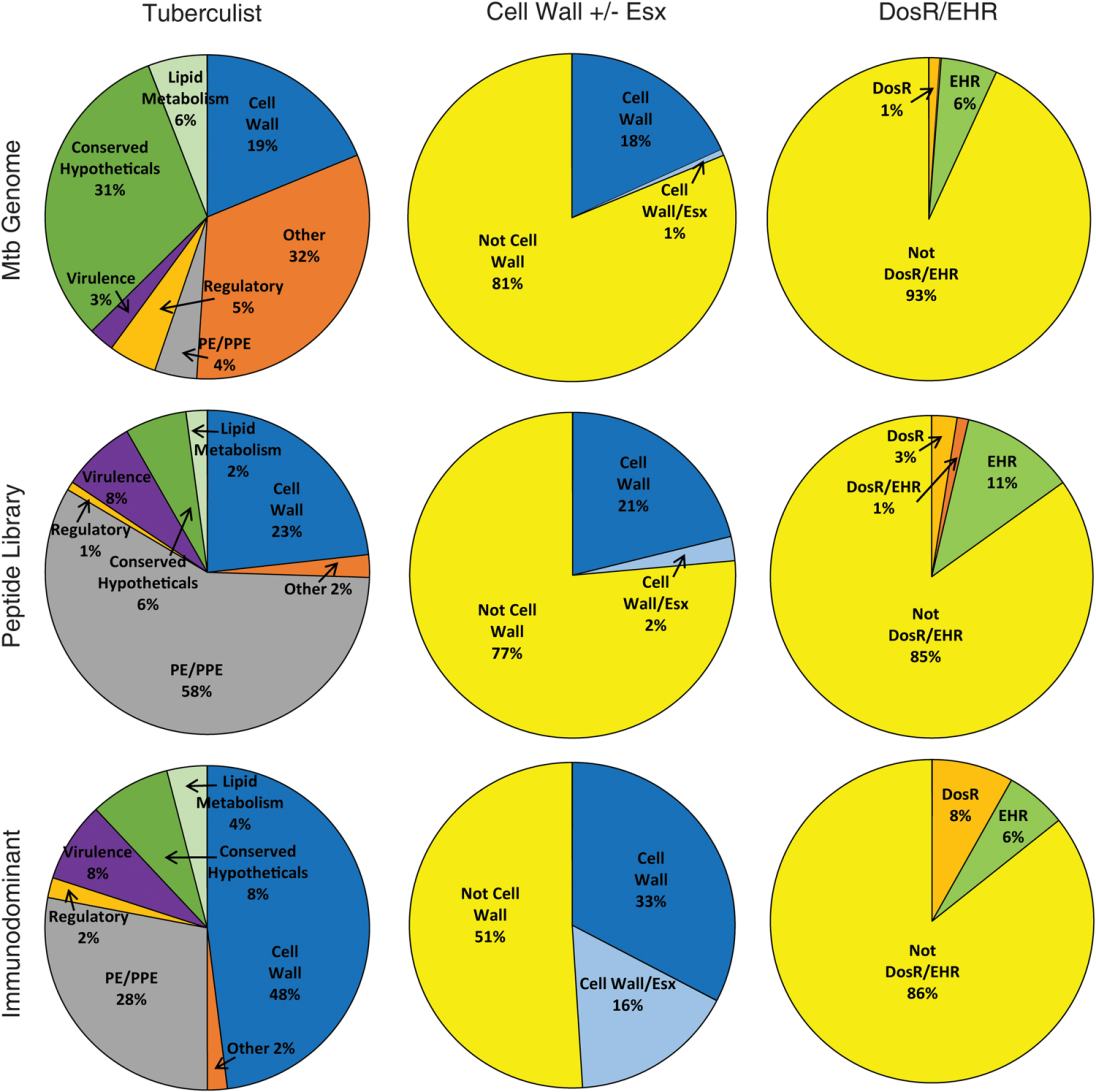
Composition of the Mtb genome, peptide library and immunodominant peptide pools according to functional classifications. For the Mtb genome, peptide library, and set of immunodominant peptide pools, the proportion of proteins categorized by Tuberculist functional classification, or categorized as an Esx family member or not, or DosR/EHR family member or not is shown. Using hypergeometric distribution, the probability of observing at most 14 peptide pools representing PE/PPE proteins out of 49 immunodominant peptide pools by chance given the high (58.8%) prevalence of peptide pools representing PE/PPE proteins in the library is 0.00098%. The probability of observing ≥ 24 peptide pools representing cell wall proteins out of 49 by chance when the prevalence in the library is 23.5%, is 0.0053%. The probability of ≥8 peptide pools representing Esx proteins out of 49 by chance when the prevalence in the library is 2.4% is <0.0001. The probability of ≥16 peptide pools representing non-Esx cell wall proteins out of 49 by chance when the prevalence in the library is 21.2% is 0.0364

As the term “immunodominant” reflects antigens commonly and strongly recognized,^22^ herein we define an immunodominant CD8 antigen as a peptide pool in the top 5% of positive responses in 3 or more of the 20 subjects tested. A total of 49 peptide pools representing part or all of 74 unique proteins met this definition of representing immunodominant antigens (Table S2). Each peptide pool represented part or all of one to three proteins and 63 (85%) proteins were represented in a single peptide pool, 10 (14%) were represented in two peptide pools, and one protein (1%) was represented by three peptide pools. The immunodominant peptide pools included one peptide pool that represented portions of two proteins belonging to distinct Tuberculist categories. Fig. 2 shows the percentage of each Tuberculist functional classification that comprises the Mtb genome, the peptide library and the set of immunodominant peptide pools. Table S3 displays data used to calculate the proportions shown in Fig. 2. As compared to the Mtb genome, the peptide library was markedly enriched for members of the PE/PPE protein families, which are comprised of abundant highly variable Mtb proteins with a proline-glutamic acid (PE) or proline-proline-glutamic acid (PPE) motif near the *N*-termini,^23^ and somewhat enriched for the genes classified as “cell wall and cell processes”. By contrast, as compared to the peptide library, cell wall proteins were enriched and the PE/PPE family proteins were relatively underrepresented among the immunodominant peptide pools. These differences between the peptide library and immunodominant peptide pools were statistically significant (Fig. 2 & Table S3). The other Tuberculist categories (other, regulatory, virulence, conserved hypotheticals, and lipid metabolism) were underrepresented in the peptide library as compared to the Mtb proteome, and either slightly enriched in (regulatory, virulence, conserved hypotheticals, and lipid metabolism) or represented a similar portion of (other) the immunodominant peptide pools as compared to peptide library. Differences for these categories between the peptide library and immunodominant peptide pools were not statistically significant. Finally, enrichment of cell wall proteins was due to both the Esx protein family, which, though it represented only 2% of the peptide library, represented 16% of the immunodominant peptide pools and also due to non-Esx cell wall proteins which were also overrepresented among the immunodominant peptide pools (Fig. 2 & Table S3).

### The CD8^+^ T cell response was not preferentially targeted towards DosR/Enduring hypoxic response (EHR) proteins

As the DosR/EHR family of proteins has been hypothesized to represent strong candidates for CD4 antigens,^24^ we analyzed these proteins separately. DosR/EHR proteins represented 15% of the peptide library. However, while some of these proteins appeared in the set of immunodominant CD8 antigens, as a class they were not overrepresented among immunodominant CD8 antigens (14%) compared to the peptide library (15%; Fig. 2 & Table S3). The immunodominant peptide pools include a total of 7 pools representing DosR and EHR proteins. Four immunodominant pools represent DosR proteins, including 3 of 5 peptide pools representing ctpF (*Rv1997*) and 1 of 3 peptide pools representing the protein encoded by *Rv1996*. Three immunodominant peptide pools represent EHR proteins, including 1 of 3 peptide pools representing the protein encoded by *Rv2557*, 1 of 3 peptide pools representing the protein encoded by *Rv2687c*, and 1 of 4 peptide pools representing PE_PGRS23 (*Rv1243c*).

### Secreted Mtb proteins are well represented among immunodominant CD8 antigens

**Fig. 3:**
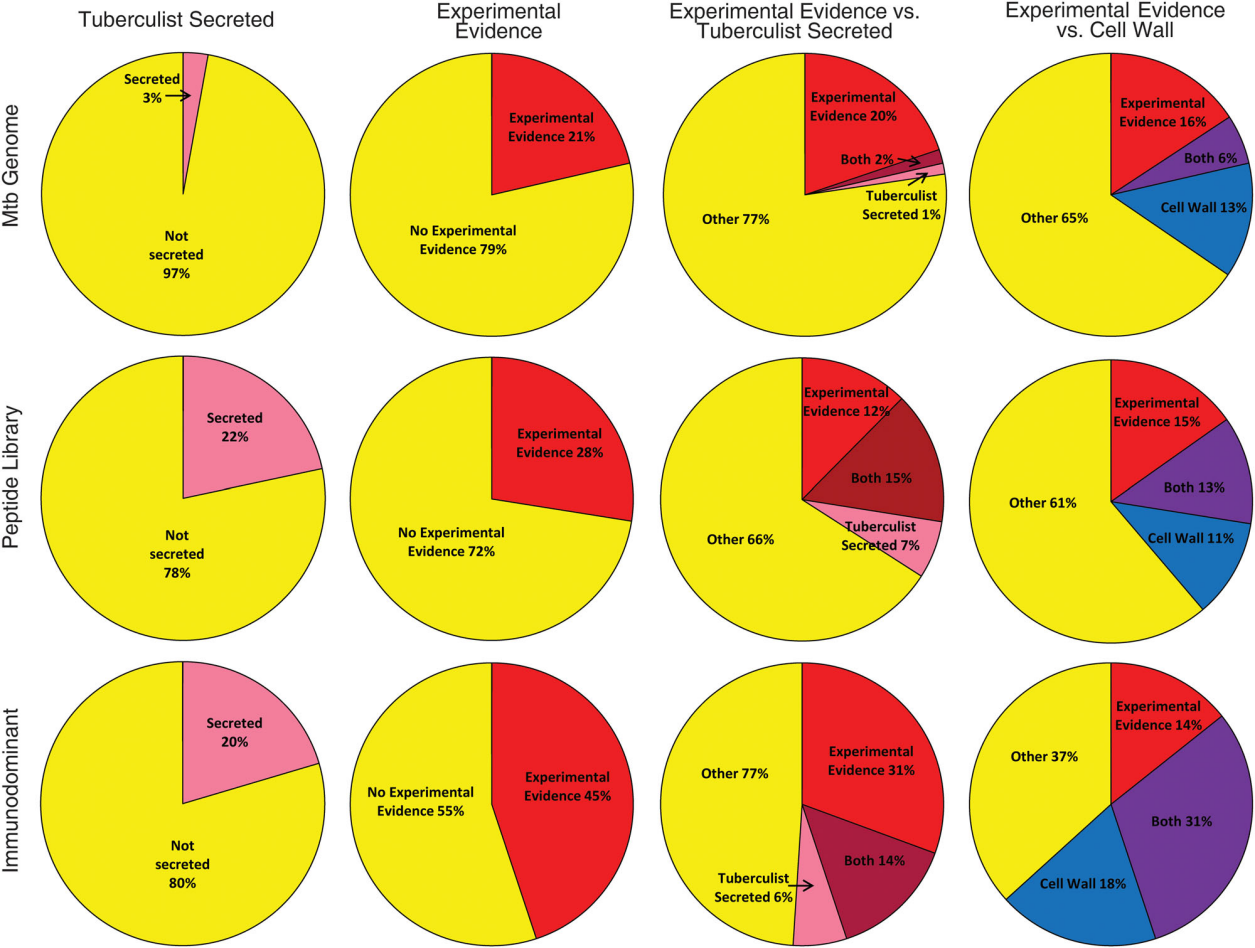
Classification of secreted proteins within the Mtb genome, peptide library and immunodominant peptide pools. For the Mtb proteome, peptide library, and set of immunodominant peptide pools, the proportions of proteins categorized as secreted as defined in Tuberculist in 2005, as secreted by an experimental evidence definition, and/or as cell wall by Tuberculist functional classification, are shown. Using hypergeometric distribution, the probability of observing ≥ 22 peptide pools representing proteins with experimental evidence of secretion out of 49 immunodominant peptide pools by chance when the prevalence of peptide pools representing proteins with experimental evidence of secretion in the library is 28%, is 0.0053%. The probability of ≥15 peptide pools representing cell wall proteins with experimental evidence of secretion out of 49 by chance when the prevalence in the library is 13% is 0.0003

Secreted Mtb proteins often represent strong CD4 antigens ^25^ and some secreted immunodominant CD4 antigens are also immunodominant CD8 antigens.^17^ Therefore, we asked if secreted proteins were overrepresented in the immunodominant peptide pools using two different definitions of “secreted”. First, all Mtb proteins listed as “secreted” in the free text field of Tuberculist (*n* = 113) were included in the peptide library and these proteins were represented in 22% of peptide library pools as compared to 3% of the Mtb genome (Fig. 3, first column & Table S4). However, similar to the DosR/EHR proteins, while a subset of these proteins were among the immunodominant peptide pools, as a class they were not overrepresented compared to the peptide library (Fig. 3, first column). Secondly, to incorporate more recent experimental evidence into a definition of secretion, we interrogated the published literature for proteins for which active secretion has been demonstrated (Table S5). Using this definition, the Mtb genome contains 856 secreted proteins representing 21% of the genome (Fig. 3, second column & Table S4). The peptide library included 217 of these proteins, which represented 28% of the peptide library. In contrast to the results using the Tuberculist definition of “secreted”, these proteins were overrepresented in the immunodominant peptide pools compared to the peptide library (45% of immunodominant peptide pools represented parts or the entirety of these Mtb proteins; Fig. 3, second column). The process for determining secretion of Mtb proteins for this second definition included accessing Tuberculist for proteins noted to be secreted in text and then further interrogating these proteins for experimental evidence of secretion. Therefore, we were able to compare the proteins designated as secreted in Tuberculist in 2005 when the peptide library was created, to the proteins designated as secreted in Tuberculist in 2014, when the interrogation of Mtb proteins for experimental evidence of secretion was performed. Of 113 proteins on the 2005 list, all but one were on the 2014 list. Of these 112 proteins, 62 (55%) met our definition of experimental evidence of secretion. Also, an additional 79 proteins were on the 2014 but not the 2005 list and all but one of these met our definition of experimental evidence of secretion (data not shown). The third column of Fig. 3 shows the percentage of proteins designated as secreted by the Tuberculist definition only, the experimental evidence of secretion definition only, or that met both definitions. Finally, as cell wall proteins frequently use the secretion machinery to reach their destinations and are sometimes shed, we also considered the overlap between cell wall proteins and proteins with experimental evidence of secretion. Cell wall proteins with experimental evidence of secretion comprised 6% of the Mtb genome, were overrepresented in the peptide library as compared to the Mtb genome (13% vs. 6%) and further overrepresented among the immunodominant peptide pools as compared to the peptide library (31 vs. 13%; Fig. 3, 4th column & Table S4).

### Composite evidence scores positively correlated with immunodominant CD8 antigens

We next sought to determine what aspect of our gene selection method, which used available genomic and proteomic information to weigh and then rank the Mtb proteins most likely to represent CD8 antigens, was predictive of finding immunodominant CD8 antigens. For this analysis, we compared the immunodominant peptide pools (representing immunodominant CD8 antigens, Table S2) to peptide pools that were not recognized by CD8^+^ T cells from any of the twenty individuals (“no response” pools, Table S6). There were 54 “no response pools” representing parts or all of 74 unique proteins. Each of these peptide pools represented parts or the entirety of one (*n* = 29), two (*n* = 26), or three (*n* = 3) proteins. Conversely, 60 proteins were fully represented by one peptide pool, 12 proteins were represented by two peptide pools, and 2 proteins were represented by three peptide pools. We then compared a Composite Evidence Score, which was calculated including weighting based upon proteomic expression, transcriptional expression, and lack of expression in BCG, as well as individual proteomic and transcriptional scores, as defined below in Methods,^21^ between the immunodominant CD8 antigen peptide pools and the “no response” pools (Table 1). For both the proteomic score and the Composite Evidence Score, scores were significantly higher for Mtb genes represented by the immunodominant peptide pools than for those represented by the “no response” peptide pools (*p* = 0.0200 and 0.0009 respectively). While the mean transcriptional expression score was higher for Mtb genes represented by the immunodominant peptide pools than by those represented by the “no response” peptide pools, this difference was not statistically significant (*p* = 0.2745).

**Table 1:** Association of peptide library gene selection method with CD8 antigenicity

	No Response			Immunodominant			Difference	Wilcoxon rank sum test *p*-value
	*N*	Mean	Std dev.	*N*	Mean	Std dev.		
Composite evidence based weight	71	1.69	3.11	70	3.92	3.80	−2.24	0.0009
Proteomic score	71	1.17	4.65	70	3.91	6.57	−2.75	0.0200
Expression score	71	3.89	7.79	70	5.36	7.67	−1.47	0.2745

### CD8 antigens defined in a racially diverse group were frequently recognized by Mtb-infected individuals from a TB endemic region

**Fig. 4:**
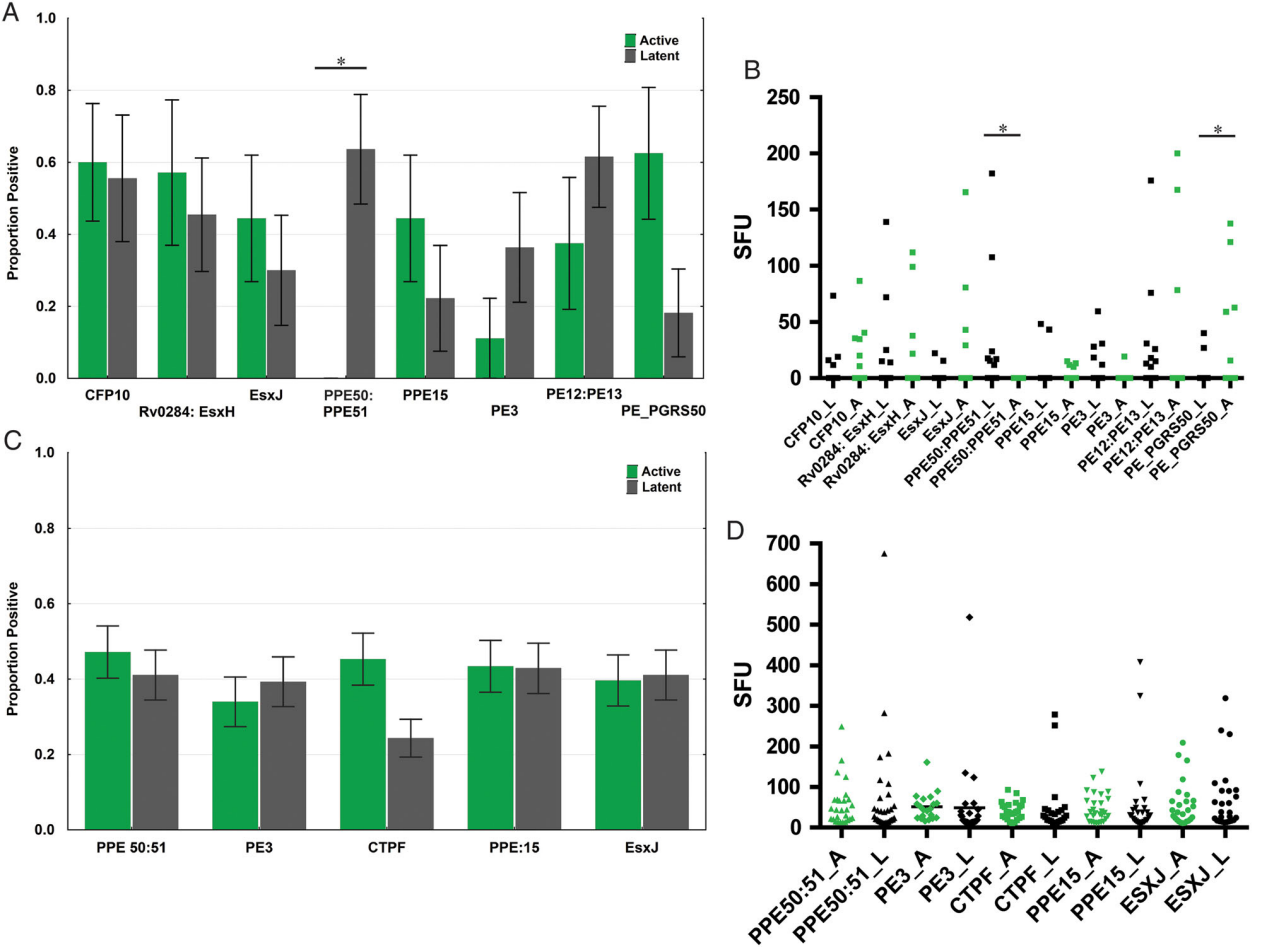
Clinical Validation of Immunodominant Peptide Pools in Mtb-infected Individuals in Kampala, Uganda. **a**, **b** IFN-γ ELISPOT was performed on CD4/CD56 depleted PBMC from approximately 10 subjects with LTBI and 10 with TB. **a** Results are shown as percent positive assays. Black bars indicate participants with LTBI and green bars indicate participants with active TB. For the PPE51:PPE50 peptide pool, the asterisk denotes that the difference in proportion positive between those with LTBI and active TB was statistically significant (*p* = 0.004, Fisher’s exact test). **b** The associated magnitude of responses reported in SFU is shown. Black dots indicate participants with LTBI and green dots indicate participants with active TB. For the PPE51:PPE50 and PE_PGRS50 peptides pools, the asterisks denote that the difference in SFU between those with LTBI and active TB were statistically significant (*p* = 0.004 and *p* = 0.024, respectively, Mann Whitney). **c**, **d** Additional validation was performed for five selected antigens. IFN-γ ELISPOT was performed on CD4 depleted PBMC on 50 subjects with LTBI and 50 with TB. **c** Results are shown stratified by disease phenotype as a percent positive by ELISPOT assays. Black bars indicate participants with LTBI and green bars indicate participants with TB. **d** The associated magnitude of responses reported in SFU is shown. Black dots indicate participants with LTBI and green dots indicate participants with active TB

Having defined a set of immunodominant CD8 antigens in a racially diverse group of Mtb-infected HIV-uninfected individuals, we next asked if these antigens were strongly and frequently recognized in individuals living in a TB endemic region, who are racially diverse from the cohorts used for antigen discovery. CD8^+^ T cells from Mtb-infected adults from Kampala, Uganda were tested for recognition of the panel of antigens represented the immundominant peptide pools. CD8^+^ T cells from approximately 20 individuals (*n* = range 17–26), comprised of both individuals with LTBI (*n* = range 8–13) and individuals with TB (*n* = range 8–16) were tested against each peptide pool. As the peptides constituting the peptide pools were 15 aa in length, to avoid detection of CD4^+^ T cell responses, we depleted CD4^+^ T cells from peripheral blood mononuclear cell (PBMC) to use as both a source of responding CD8^+^ T cells and antigen presenting cells (APC) in an IFN-γ ELISPOT assay. Examples are shown in Fig. 4a & b and all data summarized in Table S7. Of 49 pools tested, 17 were recognized by CD8^+^ T cells in at least 30% of Mtb-infected Ugandans. We conclude that strongly and commonly recognized (immunodominant) CD8 antigens defined in a racially diverse group of Mtb-infected individuals in a non-TB endemic setting were also commonly recognized in Mtb-infected individuals from a TB endemic region.

### Immunodominant CD8 antigens were recognized equivalently between individuals with LTBI compared to those with TB

A TB antigen that is differentially recognized by T cells from individuals with LTBI compared to TB disease could have value for development of TB immunodiagnostics and/or improved TB vaccines. Therefore, we tested a subset of CD8 antigens in larger cohorts of individuals (*n* = 50) with LTBI vs. TB disease, such that this comparison would be powered to detect a clinically relevant difference between the two cohorts (89% power to detect a differential in positive response rate to each antigen of 50 vs. 20%, with 5% significance level). We chose 11 peptide pools prioritizing those recognized by at least 20% of the cohorts of 20 individuals and for which there was at least a 20% difference in positive rates between groups (LTBI vs. Active TB; Table S7, grey shading, data for PPE51:PPE50 and PE_PGRS50 shown in Figs. 4a, b). Specifically, 7 of 11 peptide pools had at least a 20% different response rate between LTBI and TB disease. Representative results are shown in Figs. 4c, d. While these antigens were frequently and strongly recognized in Mtb-infected individuals, none of the antigens were differentially recognized in individuals with LTBI or active TB (i.e., *p* ≤ .05, chi-square test).

## Discussion

Despite the potential importance of CD8^+^ T cell immunity for TB vaccine development, previous CD8 antigen/epitope discovery efforts have focused on <10% of the Mtb proteome and primarily utilized HLA binding prediction algorithms.^19, 20^ Therefore we undertook a broader approach and herein report the results of the first ever HLA allele independent genome-wide CD8 antigen discovery program, defining parts or all of 74 Mtb proteins representing immunodominant antigens, only 16 of which are previously known CD8 antigens and/or proteins from which CD8 epitopes have been identified (reviewed in ref. 26). In addition, this study extends the evidence from our prior work that defined CFP10 (*Rv3874*), ESAT6 (*Rv3875*), Ag85b (*Rv1886c*), EsxG (*Rv0287*), the 19kDa protein (*Rv3763*), Mtb9.9 (*Rv1793*), as immunodominant CD8 antigens.^17^ Moreover, we validated the broad and strong recognition of these antigens defined in a racially diverse population in the United States in Mtb-infected individuals in TB endemic Uganda.

Our results demonstrate that human CD8^+^ T cells broadly target the Mtb proteome. Only 15% of the peptide pools in the library were not recognized by any of 20 ethnically diverse, Mtb-infected individuals. As the library reflected only 10% of the proteome, and was designed to reflect antigens thought to be immunogenic, it is likely that we have underestimated the proportion of the Mtb proteome that is not antigenic. Nonetheless, we speculate that this high degree of recognition of 10% of the Mtb proteome by just twenty Mtb-infected individuals suggests that the human CD8^+^ T cell response to the Mtb genome is broad and diverse. This diversity is consistent with the broad array of HLA alleles expressed worldwide. In this regard, HLA-B alleles are more diverse than HLA-A alleles, and we have shown that Mtb CD8 epitopes are preferentially presented by HLA-B alleles.^17^ In addition, our results are consistent with Tang et al, who found that 70 of 432 9-mer Mtb peptides were recognized by at least one of 41 Mtb-infected individuals.^20^ While genome-wide approaches for CD8 antigen discovery have not been performed for other large intracellular pathogens, these approaches have been described for a Dengue virus,^27^ HIV (www.hiv.lanl.gov), and CMV,^28^ in humans naturally infected with these viruses. In each case, CD8^+^ T cells responses have been found for all viral proteins. Therefore, the broad diversity of the repertoire of Mtb antigens recognized by human CD8^+^ T cells is similar to that of viruses that have been investigated. Finally, Lindestam Arlehamn *et al*. found in their genome wide approach to Mtb CD4 antigen discovery, that the human CD4^+^ T cell response is broad and diverse as well.^29^

The focusing of the T-cell response on a small set of antigens chosen from a large array of possible antigens is termed immunodominance and for antigens recognized by CD8^+^ T cells, is shaped by the efficiency with which antigens enter into the MHC-I processing pathway, the potential for peptides to bind to MHC-I molecules, and the availability of T-cell receptors (TCR) that recognize MHC class I/peptide complexes.^22^ In the outbred human population who collectively express diverse sets of HLA-A, HLA-B, and HLA-C, immunodominant antigens can only be practically defined those that are both strongly and frequently recognized in the population,^30^ as we have herein. In this regard, we found that while CD8^+^ T cell responses were diverse across different individuals, CD8^+^ T cell responses within an individual were highly focused and differed between one individual and another expressing the same HLA alleles. These results are consistent with previous studies more limited in scope including our study of HLA-B epitopes,^17^ and Tang *et al*.’s study of HLA-A2 epitopes.

Our comprehensive approach also allowed us to investigate the properties of Mtb proteins that were associated with antigenicity for the CD8^+^ T cell response. In this regard, the composite evidence based score, which reflects both functional attributes of Mtb proteins as well as evidence of expression, and a proteomic score, reflecting evidence of protein expression, were predictive of immunodominant CD8 antigens. By contrast, a score reflecting evidence of gene transcription alone was not predictive. As proteomic studies identify relatively abundantly expressed proteins, and abundance of transcripts is not entirely correlated with protein abundance due to translational regulation, these results support the conclusion that immunodominant CD8 antigens are enriched for abundantly expressed proteins. These findings for TB are consistent with that for viruses such as HIV and CMV, for which immunodominant CD8 antigens have been defined in humans. CD8 antigens, such as CMV pp65^31^ and HIV gag^32^ represent abundantly expressed proteins.

Functional attributes of Mtb proteins were associated with antigenicity as well. For example, cell wall proteins were disproportionately represented among immundominant CD8 antigens and this proportional increase was largely due to the Esx family. Esx family members have been previously described as human CD4 and CD8 antigens,^17, 33^ and in animal models are immunogenic and associated with protection.^34^ Lindestam Arlehamn *et al*. also found that human Mtb CD4 antigens were enriched among Esx family members and defined antigenic islands.^29^ By contrast we found that Esx family proteins that represent immunodominant CD8 antigens are equivalently distributed between all the *Esx* operons in Mtb.

Secreted proteins found in short-term culture filtrate^35^ represent robust CD4 antigens,^36^ some of which were protective in mouse and guinea pig vaccination models^37, 38^ and we had previously found that immunodominant CD4 antigens also represent immunodominant CD8 antigens.^17^ Using a definition for secretion that incorporated more recent experimental evidence of secretion, these proteins were disproportionately represented in the immunodominant peptide library, providing further confirmation that secreted Mtb proteins represent both robust CD4 and CD8 antigens. We speculate that secreted Mtb proteins could be preferentially processed and presented by MHC Class I.^39^

As several of the members of the PE and PPE Mtb protein families represent strong CD4 antigens,^23^ PE and PPE family members were prioritized for inclusion in the peptide library and therefore were markedly enriched in the peptide library as compared to the genome. However, in contrast to our finding with cell wall and/or secreted proteins, PE and PPE family members were relatively underrepresented among the immunodominant peptide pools. Nonetheless, parts or all of 21 PE and PPE family members were represented in the peptide pools, all of which are novel, and significant expands the repertoire of PE and PPE family members known to elicit CD8^+^ T cell responses in humans.^21^ Moreover, several of these proteins were highly immunogenic in our study of Mtb-infected Ugandan adults.

LTBI may correlate with a hypoxic microenvironment in the host, and the initial and sustained response of Mtb to hypoxia is mediated by genes encoded in the *DosR* regulon^40^ and by *EHR* genes,^41^ respectively. Therefore, the proteins encoded by *DosR* and *EHR* genes have been investigated as candidate vaccine antigens, such that these proteins could be targeted by therapeutic vaccine strategies that would prevent reactivation of Mtb from latent infection.^42^ In fact, several of these proteins are immunogenic for CD4^+^ T cells in humans with LTBI (reviewed in ref. 24). Therefore, the proteins encoded by *DosR* and *EHR* genes were prioritized for inclusion in the peptide library and while DosR and EHR proteins were not overrepresented among immunodominant CD8 antigens compared to the peptide library, DosR proteins ctpF (*Rv1997*) and *Rv1996*, and EHR proteins PE_PGRS23 (Rv1243c) and proteins encoded by *Rv2557* and *Rv2687c*, represent novel CD8 antigens. Therefore, vaccine strategies targeting DosR and EHR proteins, may consider inclusion of these antigens to broaden induction of strong CD8^+^ T cell responses.

Because of the association of *DosR* and *EHR* gene expression with a hypoxic microenvironment, which in turn may be relevant to the state of Mtb within the context of LTBI, efforts to define disease-specific Mtb antigens have focused on these genes. Indeed, several studies have demonstrated that CD4^+^ T cell responses to DosR proteins are preferentially found in individuals with LTBI compared to those with active disease.^43^ In our validation study of Mtb-infected Ugandan adults which was powered to detect differences in frequency and magnitude of CD8^+^ T cells responses between individuals with TB disease and LTBI and included two peptide pools representing DosR protein ctpF (*Rv1997*) or EHR protein PE_PGRS23 (*Rv1243c*), we did not identify disease-specific antigens. Given that CD4 antigens with disease specificity have only been described among the DosR proteins, it is possible that we did not observe disease specificity for CD8 antigens due to the small number of DosR proteins that we evaluated for this property. Nonetheless, though some CD4 antigens demonstrate evidence for disease specificity, the identification of disease-specific CD4 and CD8 antigens that would be clinically useful remains elusive.

Defining the repertoire of antigens targeted by human CD8^+^ T cells is essential to better understanding human TB immunity and has implications for the development of improved TB vaccines and diagnostics. Given the diversity and breadth of immunodominant CD4 and CD8 antigens demonstrated herein, and by Lindestam Arlehamn *et al*.,^29^ novel TB vaccines may require inclusion of several antigenic targets to provide efficacy. Moreover, TB vaccine candidates currently in clinical trials are not expected to induce CD8^+^ T cell immunity, both because these vaccines contain CD4 antigens, which are not immunodominant CD8 antigens (e.g., Ag85b) and they utilize delivery systems which are relatively inefficient in introducing antigen into the MHC Class I-restricted processing and presentation pathway (http://www.aeras.org/candidates/#candidates). Specifically, Rv3620 and Rv0288 are represented by our immunodominant peptide pools and are included in vaccine candidate ID93 expressing Rv3620 as a fusion protein with Rv2608, Rv3619, and Rv1813) and H4:IC31 expressing TB10.4 as a fusion protein with Ag85b, respectively. However, neither ID93 nor H4:IC31 are formulated to induce robust CD8^+^ T cell responses. On the other hand, the pre-clinical vaccine candidate pipeline does utilize platforms that efficiently induce CD8^+^ T cell immunity (e.g., simian adenoviruses), which would likely benefit from inclusion of immundominant CD8 antigens as we have defined herein. In addition, several pathogens for which vaccines are desperately needed, such as *Leishmania* species, *Chlamydia* species and *Plasmodium* species, also possess large genomes, making screens of peptide libraries representing the entire proteome impractical. In this regard, the success of our integrated computational and proteomic screening approach for the identification of CD8 demonstrates that this approach could also be applied to these pathogens as well. Finally, detection of Mtb-specific CD8^+^ T cells may provide clinically useful information regarding host bacillary burden in individuals with TB refs. 44, 45 and an LTBI diagnostic which measures CD8^+^ T cells has recently become commercially available (http://www.quantiferon.com/irm/content/quantiferon-tb-gold-plus.aspx?RID=412). Therefore, inclusion of additional immunodominant CD8 antigens in TB diagnostics may improve the accuracy these tests as well.

## Methods

### Study participants

Details of human subject enrollment for the ex vivo CD8^+^ T cell screens of the peptide library are described in Supplementary Methods. Individuals 18–65 years old with a history of a positive TST and/or history of TB were self-referred, informed consent was obtained, and subjects were screened for inclusion in the study which included negative HIV, HBV and HCV serologies, and acceptable levels of background of CD8^+^ T cells in an IFN-γ ELISPOT assay. In addition, for LTBI subjects, to exclude individuals with a positive TST due to exposure to BCG or atypical mycobacteria, those whose PBMC did not demonstrate Mtb-specific T cell responses were excluded. To obtain sufficient PBMC to complete the library screens, individuals underwent leukapheresis.

For clinical screening and validation studies in Uganda, individuals ≥ 18 years old with LTBI or TB disease were enrolled into a Case Western Reserve University Tuberculosis Research Unit IRB-approved study. In all cases, informed consent was obtained and individuals meeting eligibility requirements for LTBI or active TB disease were enrolled. LTBI was defined as healthy, HIV seronegative individuals with a TST ≥ 10 mm and no evidence of TB disease. TB disease was defined as HIV seronegative individuals diagnosed with pulmonary disease confirmed by sputum culture positive for Mtb. Whole blood (maximum = 50 ml) was obtained by venipuncture and used to isolate PBMC. For individuals with active TB, blood was obtained at the time of diagnosis, just prior to the initiation of therapy.

### Peptide library

Design and synthesis of the synthetic peptide library has been described elsewhere.^21^ Briefly, using available genomic and proteomic information and a weighting schema based upon a Multiple Attribute Decision Making:framework aimed to select Mtb proteins most likely to represent CD8 antigens, we designed a synthetic peptide library comprised of 15-mers overlapping by 9 aa representing the products of approximately 10% of the Mtb genome (389 of 4011 genes). Both gene product expression and function were weighted for potential to represent CD8 antigens. This included a TubercuList Functional Score calculated based upon TubercuList attributes and prioritizing genes in the “PE/PPE” family (*n* = 168) and “cell wall and cell processes” (*n* = 134) due to their potentially greater significance in the CD8^+^ T cell response. Gene products with a tuberculist functional score ≥ 10 were included in the library (*n* = 256) and included genes from the functional categories: conserved hypotheticals (*n* = 30) ; virulence, detoxification, adaptation (*n* = 29), intermediary metabolism and respiration (*n* = 10), lipid metabolism (*n* = 9), regulatory proteins (*n* = 5), and conserved hypotheticals with an orthologue in *M. bovis* (*n* = 4). Also a Composite Evidence Score was calculated, which included additional weighting based upon proteomic expression, transcriptional expression, and lack of expression in BCG. Proteomic data included cell-associated proteins from the Erdmann strain of Mtb and secreted and cell-associated proteins from H37Rv Mtb. Proteins detected as secreted were given a greater weighting than cell-associated gene products. For transcriptional profiling of Mtb gene expression in macrophages,^46^ induced expression associated with adaptation to intracellular growth or stress was given a greater weighting than constitutively expressed Mtb genes. Scores for proteomic expression, transcriptional expression, and lack of expression in BCG were added to the Tuberculist score and averaged to obtain the composite evidence score. Gene products with a composite evidence score ≥ 5.33 were included in the library (*n* = 91). Finally, genes of special interest that were not selected based upon the tuberculist functional score or the composite evidence score were added and were comprised of previously described CD8 antigens (*n* = 10), or CD4 antigens (*n* = 9), or genes annotated as “ESAT-like” (*n* = 15) or as “secreted (*n* = 8) in Tuberculist. A total of 39,499 peptides were synthesized (Jerini Peptide Technologies, Berlin Germany) representing the 389 selected gene products and pooled into 789 pools (50 peptides/pool) in a 96 well format in a total of nine plates. Five blank wells and one well of an irrelevant peptide pool representing SIV Gag, were included on each of the nine plates.

### Definition of secreted Mtb proteins based upon experimental evidence

Proteomics studies cited by Tuberculist were compiled and Rv numbers of genes in each study for which there was protein expression consistent with secretion were tabulated. In addition, the PubMed reference database was searched for “tuberculosis secreted protein” and publications from 1997 to 2014 were tabulated and curated for experimental evidence of secretion. Next, all Rv numbers denoted as “secreted” in the free text field of Tuberculist accessed July, 2014, were tabulated and cross-referenced with proteomic studies and PubMed reference lists. Then all proteins that were only on the Tuberculist “secreted” list were searched individually in PubMed and curated for experimental evidence of secretion.

### Generation and infection of peripheral blood derived DC

Monocyte-derived DC were prepared according to a modified method of Romani *et al*.^10^. To generate Mtb-infected DC, day 5 DC (1 × 10^6^/well) were cultured overnight in the presence of Mtb at a multiplicity of infection = 30:1. As heavy infection is required to optimize entry of antigen into the class I processing pathway,^12^ we have determined that this multiplicity of infection is optimal for detection of Mtb-specific CD8^+^ T cells. After 18 h, the cells were harvested and resuspended in RPMI/10% human serum.

### Isolation of CD8^+^ T cells

CD8^+^ T cells were positively selected from cryopreserved PBMC using CD8 microbeads (Miltenyi Biotec, Auburn, CA, USA) using the standard protocol. Resulting cell populations contained >99% CD8^+^ T cells as determined by CD8 expression by flow cytometry.

### Ex vivo CD8^+^ T cell screen of peptide library

CD8^+^ T cells from 20 donors were tested once for their response to autologous DC. Each plate of the genomic peptide library was screened in duplicate, for a total of 18 ELISPOT plates per screen. CD8^+^ T cells (250,000 cells/ well), autologous DCs (20,000 cells/well), and IL-2 (0.5 ng/ml) were added to peptide (final 5 ug/ml, individual peptides) in the ELISPOT plates. Five media control wells were included on each plate. Spots were enumerated using the AID EliSpot Reader System (Strassberg, Germany).

### Ex vivo CD8^+^ T cell IFN-γ ELISPOT assay

IFN-γ ELISPOT assays on Ugandan samples were performed as described previously.^44^ Briefly, CD8^+^ T cells and APC were negatively selected from PBMC by one of two methods. From 2004 to 2008, cryopreserved PBMC were thawed and then depleted of CD4^+^ and CD56^+^ cells by magnetic bead separation (Miltenyi Biotec, Auburn, CA, USA). We determined empirically a priori that CD56+ cell depletion reduced the background in the assay. From 2009 to 2012, fresh whole blood was depleted of CD4^+^ cells using RosetteSep technology from StemCell Technologies. Using RosetteSep, we determined empirically a priori that CD56+ depletion was not necessary to eliminate background in the assay. CD4-depleted PBMC (250,000 cells per well) were then used as the source of responding CD8^+^ T cells and APC in an IFN-γ ELISPOT assay, using overlapping synthetic peptide pools consisting of 15-mers overlapping by 11 aa (final concentration 5 μg/ml) as a source of antigen. The ELISPOT assay was performed once on each donor. All determinations were performed in duplicate. Positive responses were defined as those that are 2SD above the media control, and greater than 10 SFU, which is a similar but more conservative rule than we have utilized previously in cohorts of young Ugandan children.^44^

### Data analysis

For the ex vivo CD8^+^ T cell screen of peptide library, a well was scored positive if the SFU, less the mean of the media wells, was greater than or equal to ten and the SFU was greater than or equal to twice the mean of the media ^47^. For each plate, the mean of the five media control wells was subtracted from each well of that plate to normalize between plates. The average SFU was computed as follows: if both technical replicates were positive then the average of the adjusted spot counts represented a true average, otherwise it represented the adjusted spot count for the positive technical replicate. Each technical replicate on each plate was then scored.

We define an immunodominant CD8 antigen as a peptide pool which was in the top 5% of positive responses in 3 or more of the 20 subjects tested. Since distributions of adjusted spot counts varied greatly between subjects, conventional approaches such as determination of average spot counts for identifying a strong response would be strongly affected by individual subjects with high magnitude responses, thus biasing the analysis. Therefore, we adopted a ranks based procedure. First, for each individual, adjusted spot counts were ranked by their magnitude and the top 5% of peptide pools with positive responses were identified. Then the peptide pools which were present in the top 5% of responses in three or more of twenty subjects were identified as immunodominant peptide pools. To compare the magnitude of SFU between active TB and LTBI donors, a non-parametric test (Mann–Whitney test) was used, because the distributions of the data were positive skewed due to numerous values near zero. Functional categories for these selected peptide pools were summarized using tables (Tables S3 & S4). If a peptide pool contained two genes that belonged to two distinct functional categories, then each functional category was counted separately. Pie charts were used to visualize distribution of functional classifications for specific families of proteins. Hypergeometric probabilities were calculated to estimate the prevalence of specific families of proteins, including Esx, among immunodominant antigens. The Wilcoxon rank sum test was used to compare proteomic, transcriptional expression, and the composite evidence based scores between “immunodominant CD8 antigen” pools and the “no response” pools.

For the clinical screening and validation studies in Uganda, separately for each antigen, we use a chi-square test/ Fisher’s exact test to compare a difference in the positive response rates between TB and LTBI. For the validation studies, the sample size of 50 TB and 50 LTBI subjects provided an 85% power to detect a differential in positive response rate to each antigen of 50 vs. 20%, with 5% significance level, using a two-sided Fisher’s Exact test. We estimated the prevalence of positive response for TB subjects and separately for LTBI with as small as 14.5% margin of error (i.e., a half the width of the 95% exact confidence interval). To compare the magnitude of SFU between active TB and LTBI donors a non-parametric test, (Mann–Whitney test) was used, because the data was not normally distributed.

### Ethics approvals

The Oregon Health & Science University IRB (Portland, Oregon), the University Hospitals/Case Medical Center IRB (Cleveland, Ohio) and the Joint Clinical Research Centre IRB (Kampala, Uganda) approved this study. Written, informed consent was obtained from all participants before enrollment.

## Electronic supplementary material

Supplementary Figure S1

Supplementary Table S1

Supplementary Table S2

Supplementary Table S3

Supplementary Table S4

Supplementary Table S5

Supplementary Table S6

Supplementary Table S7

Supplementary Information

Supplementary Methods
