# Supplementary figures and images for: Comprehensive definition of human immunodominant CD8 antigens in tuberculosis
Source: NPJ Vaccines. 2017 Apr 3;2:8. doi: 10.1038/s41541-017-0008-6 (PMC5538316; doi:10.1038/s41541-017-0008-6)

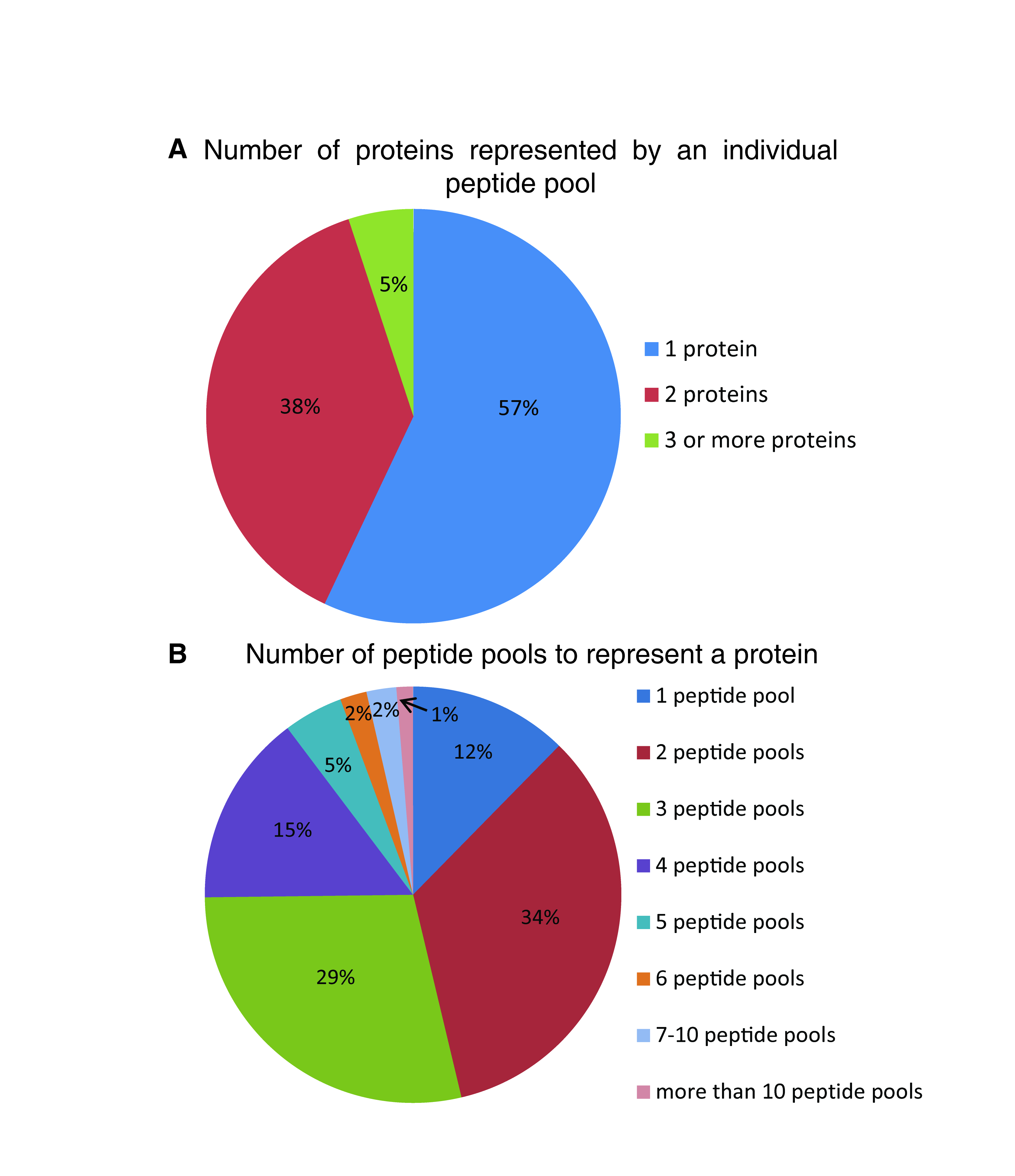

Supplement: Supplementary file 1 — Supplementary Figure S1 [file 41541_2017_8_MOESM1_ESM.tif]
