# Supplementary material for: Comprehensive definition of human immunodominant CD8 antigens in tuberculosis
Source: NPJ Vaccines. 2017 Apr 3;2:8. doi: 10.1038/s41541-017-0008-6 (PMC5538316; doi:10.1038/s41541-017-0008-6)
Supplement: Supplementary file 2 — Supplementary Table S1 [file 41541_2017_8_MOESM2_ESM.docx]

### Table S1. Subject enrollment for peptide library screens

|  | Goal | Screened^1^ | Apheresed^2^ | Successful donor screens |
| --- | --- | --- | --- | --- |
| LTBI Caucasian | 5 | 21 | 7 | 6 |
| LTBI SE Asian | 5 | 16 | 5 | 5 |
| LTBI African American | 5 | 28 | 9 | 4 |
| Active TB | 5 | 6 | 6 | 5 |
| Total | 20 | 71 | 27 | 20 |
| ^1^Of the 44 individuals who failed screening, 10 individuals demonstrated unacceptable background of CD8^+^ T cells in the IFN-γ ELISPOT assay, and 34 individuals with a self-reported positive TST, did not demonstrate CD4^+^ T cell responses to ESAT6 or CFP10 in PBMC, which may be due to false positive TST results, the reasons for which include prior BCG immunization, exposure to environmental non-tuberculosis mycobacteria, or inappropriate TST placement. While not criteria for a successful screen, all 5 active TB subjects demonstrated ESAT6 or CFP10-specific T cell responses in PBMC.  ^2^For subjects who were apheresed but who had failed donor screens, there was insufficient leukapheresis product to complete the library screen (*n* =5) or the library screen was not interpretable due to CD8^+^ T cell background (*n* = 2). | | | | |
