# Supplementary material for: Comprehensive definition of human immunodominant CD8 antigens in tuberculosis
Source: NPJ Vaccines. 2017 Apr 3;2:8. doi: 10.1038/s41541-017-0008-6 (PMC5538316; doi:10.1038/s41541-017-0008-6)
Supplement: Supplementary file 3 — Supplementary Table S2 [file 41541_2017_8_MOESM3_ESM.docx]

**Table S2. Immunodominant peptide pools**

| **Rv Number**  **(# peptides in pool)^1^** | **Name^1^** | **Tuberculist Category** |
| --- | --- | --- |
| *Rv0383c*(30) : *Rv0394c*(20) | *Rv0383c* : *Rv0394c* | cell wall and cell processes |
| *Rv3163c*(41) : *Rv3194c*(9) | *Rv3163c* : *Rv3194c* | cell wall and cell processes |
| *Rv3875*(21) : *Rv3666c*(19) : *Rv0259c*(10) | *esxA* : *dppA* : *Rv0259c* | cell wall and cell processes : cell wall and cell processes : conserved hypotheticals |
| *Rv2542*(39) : *Rv2393*(11) | *Rv2542* : *Rv2393* | conserved hypotheticals |
| *Rv2672*(38) : *Rv2780*(12) | *Rv2672* : *ald* | intermediary metabolism and respiration |
| *Rv0129c*(30) : *Rv1886c*(23) | *fbpC* : *fbpB* | lipid metabolism |
| *Rv0594*(50) | *mce2F* | virulence, detoxification, adaptation |
| *Rv0594*(45) : *Rv1966*(5) | *mce2F* : *mce3A* | virulence, detoxification, adaptation |
| *Rv1966*(49) : *Rv1967*(1) | *mce3A* : *mce3B* | virulence, detoxification, adaptation |
| *Rv1969*(35) : *Rv1971*(15) | *mce3D* : *mce3F* | virulence, detoxification, adaptation |
| *Rv0284*(50) | *Rv0284* | cell wall and cell processes |
| *Rv0287*(22) : *Rv0284*(17) : *Rv0288*(11) | *esxG* : *Rv0284* : *esxH* | cell wall and cell processes |
| *Rv0446c*(39) : *Rv0288*(11) | *Rv0446c* : *esxH* | cell wall and cell processes |
| *Rv1072*(26) : *Rv1038c*(22) : *Rv1037c*(2) | *Rv1072* : *esxJ* : *esxI* | cell wall and cell processes |
| *Rv1132*(33) : *Rv1184c*(17) | *Rv1132* : *Rv1184c* | cell wall and cell processes |
| *Rv1197*(22) : *Rv1184c*(20) : *Rv1198*(8) | *esxK* : *Rv1184c* : *esxL* | cell wall and cell processes |
| *Rv1739c*(34) : *Rv1793*(16) | *Rv1739c* : *esxN* | cell wall and cell processes |
| *Rv1979c*(50) | *Rv1979c* | cell wall and cell processes |
| *Rv1980c*(28) : *Rv1984c*(22) | *mpt64* : *cfp21* | cell wall and cell processes |
| *Rv1984c*(30) : *Rv1986*(20) | *cfp21* : *Rv1986* | cell wall and cell processes |
| *Rv1992c*(40) : *Rv1987*(10) | *ctpG* : *Rv1987* | cell wall and cell processes |
| *Rv1992c*(50) | *ctpG* | cell wall and cell processes |
| *Rv1997*(50) | *ctpF* | cell wall and cell processes |
| *Rv1997*(50) | *ctpF* | cell wall and cell processes |
| *Rv1997*(50) | *ctpF* | cell wall and cell processes |
| *Rv2041c*(50) | *Rv2041c* | cell wall and cell processes |
| *Rv2041c*(43) : *Rv2093c*(7) | *Rv2041c* : *tatC* | cell wall and cell processes |
| *Rv2270*(32) : *Rv2093c*(18) | *lppN* : *tatC* | cell wall and cell processes |
| *Rv2686c*(44) : *Rv2687c*(6) | *Rv2686c* : *Rv2687c* | cell wall and cell processes |
| *Rv3641c*(33) : *Rv3620c*(17) | *fic* : *esxW* | cell wall and cell processes |
| *Rv3874*(23) : *Rv3763*(18) : *Rv3877*(9) | *esxB* : *lpqH* : *Rv3877* | cell wall and cell processes |
| *Rv1996*(50) | *Rv1996* | conserved hypotheticals |
| *Rv3555c*(48) : *Rv2557*(2) | *Rv3555c* : *Rv2557* | conserved hypotheticals : conserved hypotheticals with an orthologue in M. bovis |
| *Rv0824c*(50) | *desA1* | lipid metabolism |
| *Rv0151c*(50) | *PE1* | PE/PPE |
| *Rv0159c*(50) | *PE3* | PE/PPE |
| *Rv1039c*(50) : *Rv2768c*(1) | *PPE15* : *PPE43* | PE/PPE |
| *Rv1172c*(32) : *Rv1195*(18) | *PE12* : *PE13* | PE/PPE |
| *Rv1243c*(50) | *PE_PGRS23* | PE/PPE |
| *Rv1706c*(50) | *PPE23* | PE/PPE |
| *Rv1788*(22) : *Rv1787*(17) : *Rv1789*(11) | *PE18* : *PPE25* : *PPE26* | PE/PPE |
| *Rv1983*(36) : *Rv1918c*(14) | *PE_PGRS35* : *PPE35* | PE/PPE |
| *Rv3345c*(50) | *PE_PGRS50* | PE/PPE |
| *Rv3347c*(50) | *PPE55* | PE/PPE |
| *Rv3347c*(50) | *PPE55* | PE/PPE |
| *Rv3136*(46) : *Rv3135*(4) | *PPE51* : *PPE50* | PE/PPE |
| *Rv3514*(47) : *Rv3532*(3) | *PE_PGRS57* : *PPE61* | PE/PPE |
| *Rv3558*(44) : *Rv3539*(6) | *PPE64* : *PPE63* | PE/PPE |
| *Rv2711*(37) : *Rv1404*(13) | *ideR* : *Rv1404* | regulatory proteins |

^1^The gray shading denotes previously known CD8 antigens, or proteins from which CD8 epitopes have been identified.
