# Supplementary material for: Comprehensive definition of human immunodominant CD8 antigens in tuberculosis
Source: NPJ Vaccines. 2017 Apr 3;2:8. doi: 10.1038/s41541-017-0008-6 (PMC5538316; doi:10.1038/s41541-017-0008-6)
Supplement: Supplementary file 4 — Supplementary Table S3 [file 41541_2017_8_MOESM4_ESM.docx]

**Table S3. Data used to calculate proportions for pie charts displayed in Figure 2.**

| Tuberculist Functional Categories (TFC) | | |  |  |  |  |  |  |
| --- | --- | --- | --- | --- | --- | --- | --- | --- |
| Mtb Genome | | | Peptide Library (789 peptide pools) | | | Immunodominant (49 peptide pools) | | |
| TFC | Freq | % | TFC | Freq^1^ | % | TFC | Freq^1^ | % |
| Cell Wall | 751 | 18.78 | Cell Wall | 186 | 23.25 | Cell Wall | 24 | 48 |
| Other | 1290 | 32.26 | Other | 18 | 2.25 | Other | 1 | 2 |
| PE/PPE | 168 | 4.2 | PE/PPE | 464 | 58 | PE/PPE | 14 | 28 |
| Regulatory | 192 | 4.8 | Regulatory | 7 | 0.88 | Regulatory | 1 | 2 |
| Virulence | 102 | 2.55 | Virulence | 59 | 7.38 | Virulence | 4 | 8 |
| conserved hypotheticals | 1259 | 31.48 | conserved hypotheticals | 49 | 6.13 | conserved hypotheticals | 4 | 8 |
| lipid metabolism | 237 | 5.93 | lipid metabolism | 17 | 2.13 | lipid metabolism | 2 | 4 |
| Total | 3999 | 100 |  | 800 | 100 |  | 50 | 100 |
|  |  |  |  |  |  |  |  |  |
| Cell Wall +/- ESX |  |  |  |  |  |  |  |  |
| Mtb Genome | | | Peptide Library | | | Immunodominant | | |
| Cell Wall/ESX | Freq | % | Cell Wall/ESX | Freq | % | Cell Wall/ESX | Freq | % |
| Cell Wall | 728 | 18.2 | Cell Wall | 167 | 21.17 | Cell Wall | 16 | 32.65 |
| Both Cell Wall & ESX | 23 | 0.58 | Both Cell Wall & ESX | 19 | 2.41 | Both Cell Wall & ESX | 8 | 16.33 |
| Not Cell Wall | 3248 | 81.22 | Not Cell Wall | 603 | 76.43 | Not Cell Wall | 25 | 51.02 |
| Total | 3999 | 100 | Total | 789 | 100 | Total | 49 | 100 |
|  |  |  |  |  |  |  |  |  |
| DosR/EHR |  |  |  |  |  |  |  |  |
| Mtb Genome | | | Peptide Library | | | Immunodominant | | |
| DOSR/EHR | Freq | % | DOSR/EHR | Freq | % | DOSR/EHR | Freq | % |
| DosR | 44 | 1.1 | DosR | 20 | 2.53 | DosR | 4 | 8.16 |
| Both DosR & EHR | 5 | 0.13 | Both DosR & EHR | 9 | 1.14 | Both DosR & EHR | 0 | 0 |
| EHR | 225 | 5.63 | EHR | 90 | 11.41 | EHR | 3 | 6.12 |
| Not DosR or EHR | 3725 | 93.15 | Not DosR or EHR | 670 | 84.92 | Not DosR or EHR | 42 | 85.71 |
| Total | 3999 | 100 | Total | 789 | 100 | Total | 49 | 100 |

^1^ If a peptide pool contained two genes that belonged to two distinct functional categories, then each functional category was counted separately.
