# Supplementary material for: Comprehensive definition of human immunodominant CD8 antigens in tuberculosis
Source: NPJ Vaccines. 2017 Apr 3;2:8. doi: 10.1038/s41541-017-0008-6 (PMC5538316; doi:10.1038/s41541-017-0008-6)
Supplement: Supplementary file 5 — Supplementary Table S4 [file 41541_2017_8_MOESM5_ESM.docx]

**Table S4. Data used to calculate proportions for pie charts displayed in Figure 3.**

| **Tuberculist Secreted** | | |  |  |  |  |  |  |
| --- | --- | --- | --- | --- | --- | --- | --- | --- |
| Mtb Genome | | | Peptide Library (789 peptide pools) | | | Immunodominant (49 peptide pools) | | |
| Tuberculist secreted | Freq | % | Tuberculist secreted | Freq | % | Tuberculist secreted | Freq | % |
| Secreted | 113 | 2.83 | Secreted | 171 | 21.67 | Secreted | 10 | 20.41 |
| Not secreted | 3886 | 97.17 | Not secreted | 618 | 78.33 | Not secreted | 39 | 79.59 |
| Total | 3999 | 100.00 | Total | 789 | 100.00 | Total | 49 | 100.00 |
|  |  |  |  |  |  |  |  |  |
| **Experimental Evidence for Secretion** | | |  |  |  |  |  |  |
| Mtb Genome | | | Peptide Library | | | Immunodominant | | |
| Experimental Evidence for Secretion | Freq | % | Experimental Evidence for Secretion | Freq | % | Experimental Evidence for Secretion | Freq | % |
| Experimental Evidence for Secretion | 856 | 21.41 | Experimental Evidence for Secretion | 217 | 27.50 | Experimental Evidence for Secretion | 22 | 44.90 |
| No Experimental Evidence for Secretion | 3143 | 78.59 | No Experimental Evidence for Secretion | 572 | 72.50 | No Experimental Evidence for Secretion | 27 | 55.10 |
| Total | 3999 | 100.00 | Total | 789 | 100.00 | Total | 49 | 100.00 |
|  |  |  |  |  |  |  |  |  |
| **Experimental Evidence vs. Tuberculist Secreted** | | | | | |  |  |  |
| Mtb Genome | | | Peptide Library | | | Immunodominant | | |
| Experimental Evidence for Secretion vs. Tuberculist Secreted | Freq | % | Experimental Evidence for Secretion vs. Tuberculist Secreted | Freq | % | Experimental Evidence for Secretion vs. Tuberculist Secreted | Freq | % |
| Experimental Evidence for Secretion | 793 | 19.83 | Experimental Evidence for Secretion | 98 | 12.42 | Experimental Evidence for Secretion | 15 | 30.61 |
| Experimental Evidence & Tuberculist Secreted | 63 | 1.58 | Experimental Evidence & Tuberculist Secreted | 119 | 15.08 | Experimental Evidence & Tuberculist Secreted | 7 | 14.29 |
| Tuberculist Secreted | 50 | 1.25 | Tuberculist Secreted | 52 | 6.59 | Tuberculist Secreted | 3 | 6.12 |
| Not secreted | 3093 | 77.34 | Not secreted | 520 | 65.91 | Not secreted | 24 | 48.98 |
| Total | 3999 | 100.00 | Total | 789 | 100.00 | Total | 49 | 100.00 |
|  |  |  |  |  |  |  |  |  |
|  |  |  |  |  |  |  |  |  |
|  |  |  |  |  |  |  |  |  |
|  |  |  |  |  |  |  |  |  |
| **Experimental Evidence for Secretion vs. Cell Wall** | | | | | | | | |
| Mtb Genome | | | Peptide Library | | | Immunodominant | | |
| Experimental Evidence for Secretion vs. Cell Wall | Freq | % | Experimental Evidence for Secretion vs. Cell Wall | Freq | % | Experimental Evidence for Secretion vs. Cell Wall | Freq | % |
| Experimental Evidence  for Secretion | 630 | 15.75 | Experimental Evidence  for Secretion | 120 | 15.21 | Experimental Evidence  for Secretion | 7 | 14.92 |
| Experimental Evidence for Secretion & Cell Wall | 226 | 5.65 | Experimental Evidence for Secretion & Cell Wall | 97 | 12.29 | Experimental Evidence for Secretion & Cell Wall | 15 | 30.61 |
| Cell Wall | 525 | 13.13 | Cell Wall | 89 | 11.28 | Cell Wall | 9 | 18.37 |
| Neither Experimental Evidence for Secrtion or Cell Wall | 2618 | 65.47 | Neither Experimental Evidence for Secrtion or Cell Wall | 483 | 61.22 | Neither Experimental Evidence for Secrtion or Cell Wall | 18 | 36.73 |
| Total | 3999 | 100 | Total | 789 | 100 | Total | 49 | 100 |
