# Supplementary material for: Comprehensive definition of human immunodominant CD8 antigens in tuberculosis
Source: NPJ Vaccines. 2017 Apr 3;2:8. doi: 10.1038/s41541-017-0008-6 (PMC5538316; doi:10.1038/s41541-017-0008-6)
Supplement: Supplementary file 6 — Supplementary Table S5 [file 41541_2017_8_MOESM6_ESM.docx]

**Table S5. Experimental Evidence for Secretion**

| **Rv Number:** | **Reference** | **Rv Number:** | **Reference** | **Rv Number:** | **Reference** | **Rv Number:** | **Reference** |
| --- | --- | --- | --- | --- | --- | --- | --- |
| *Rv0001* | 4 | *Rv0838* | 2,3 | *Rv1860* | 1,2,3,19 | *Rv2935* | 4 |
| *Rv0002* | 2 | *Rv0848* | 4 | *Rv1867* | 4 | *Rv2938* | 1 |
| *Rv0005* | 4 | *Rv0851c* | 3 | *Rv1869c* | 2,3 | *Rv2940c* | 2,4 |
| *Rv0009* | 2,5 | *Rv0852* | 2 | *Rv1872c* | 4 | *Rv2941* | 2 |
| *Rv0012* | 3 | *Rv0858c* | 2 | *Rv1876* | 2 | *Rv2945c* | 1,2,3,4 |
| *Rv0014c* | 2 | *Rv0860* | 2 | *Rv1878* | 2 | *Rv2967c* | 4 |
| *Rv0015c* | 3 | *Rv0861c* | 4 | *Rv1881c* | 4 | *Rv2969c* | 2,28 |
| *Rv0019c* | 3 | *Rv0862c* | 4 | *Rv1884c* | 2,3,13 | *Rv2971* | 2 |
| *Rv0020c* | 2 | *Rv0866* | 2 | *Rv1885c* | 2,20 | *Rv2974c* | 4 |
| *Rv0022c* | 4 | *Rv0867c* | 2,3,13 | *Rv1886c* | 1,2,3,7 | *Rv2978c* | 4 |
| *Rv0032* | 4 | *Rv0869c* | 4 | *Rv1887* | 1 | *Rv2986c* | 4 |
| *Rv0040c* | 2,3,6 | *Rv0876c* | 1 | *Rv1891* | 1,2,3 | *Rv2988c* | 4 |
| *Rv0041* | 2,4 | *Rv0884c* | 2 | *Rv1899c* | 3 | *Rv2991* | 2 |
| *Rv0046c* | 2 | *Rv0888* | 2 | *Rv1906c* | 2,3 | *Rv2992c* | 2 |
| *Rv0050* | 2 | *Rv0889c* | 2 | *Rv1908c* | 2 | *Rv2994* | 3 |
| *Rv0054* | 2 | *Rv0890c* | 4 | *Rv1910c* | 2,3 | *Rv2995c* | 2 |
| *Rv0056* | 4 | *Rv0895* | 4 | *Rv1911c* | 2,3 | *Rv2999* | 4 |
| *Rv0058* | 4 | *Rv0896* | 2 | *Rv1922* | 2 | *Rv3001c* | 2 |
| *Rv0062* | 2,3 | *Rv0899* | 2 | *Rv1925* | 4 | *Rv3004* | 3 |
| *Rv0063* | 2,3 | *Rv0903c* | 2 | *Rv1926c* | 2,3,15 | *Rv3006* | 2,3 |
| *Rv0064* | 1,3 | *Rv0905* | 2,4 | *Rv1932* | 2,18 | *Rv3010c* | 2,4 |
| *Rv0066c* | 2 | *Rv0907* | 2 | *Rv1934c* | 4 | *Rv3014c* | 4 |
| *Rv0072* | 1 | *Rv0913c* | 2 | *Rv1938* | 2 | *Rv3016* | 2,3 |
| *Rv0075* | 2 | *Rv0920c* | 4 | *Rv1980c* | 2,3,21 | *Rv3028c* | 2,4 |
| *Rv0078A* | 2 | *Rv0928* | 2,3 | *Rv1981c* | 2 | *Rv3033* | 2,3 |
| *Rv0079* | 4 | *Rv0931c* | 1 | *Rv1984c* | 1,2,3,18 | *Rv3034c* | 4 |
| *Rv0088* | 4 | *Rv0932c* | 2,3 | *Rv1987* | 2 | *Rv3036c* | 1,2,3 |
| *Rv0089* | 4 | *Rv0934* | 1,2,3,4,7 | *Rv1989c* | 2 | *Rv3044* | 2,3 |
| *Rv0092* | 1 | *Rv0946c* | 2 | *Rv1996* | 4 | *Rv3045* | 2,4 |
| *Rv0101* | 4 | *Rv0949* | 4 | *Rv1997* | 4 | *Rv3048c* | 2 |
| *Rv0107c* | 4 | *Rv0951* | 2,4 | *Rv2000* | 4 | *Rv3049c* | 4 |
| *Rv0111* | 4 | *Rv0952* | 4 | *Rv2004c* | 4 | *Rv3050c* | 4 |
| *Rv0116c* | 1,2,3 | *Rv0957* | 2 | *Rv2005c* | 4 | *Rv3060c* | 4 |
| *Rv0120c* | 4 | *Rv0958* | 4 | *Rv2006* | 2,4 | *Rv3061c* | 2,4 |
| *Rv0122* | 4 | *Rv0982* | 3 | *Rv2030c* | 2 | *Rv3062* | 4 |
| *Rv0125* | 1,2,3,7 | *Rv0983* | 1,2 | *Rv2031c* | 2,4 | *Rv3067* | 2 |
| *Rv0126* | 2 | *Rv0984* | 2 | *Rv2035* | 2 | *Rv3080c* | 4 |
| *Rv0127* | 2 | *Rv0988* | 2 | *Rv2040c* | 1 | *Rv3089* | 2 |
| *Rv0129c* | 2,3 | *Rv0996* | 3 | *Rv2048c* | 4 | *Rv3090* | 1 |
| *Rv0139* | 4 | *Rv0999* | 2,3 | *Rv2051c* | 4 | *Rv3096* | 4 |
| *Rv0147* | 4 | *Rv1001* | 2 | *Rv2052c* | 4 | *Rv3097c* | 29 |
| *Rv0148* | 2 | *Rv1004c* | 1 | *Rv2055c* | 4 | *Rv3101c* | 4,7 |
| *Rv0153c* | 8 | *Rv1007c* | 2 | *Rv2056c* | 4 | *Rv3103c* | 1 |
| *Rv0164* | 2,3 | *Rv1009* | 1,13 | *Rv2059* | 4 | *Rv3106* | 3 |
| *Rv0169* | 1 | *Rv1013* | 4 | *Rv2060* | 3 | *Rv3127* | 4 |
| *Rv0170* | 1,3 | *Rv1016c* | 2 | *Rv2061c* | 4 | *Rv3130c* | 4 |
| *Rv0171* | 1 | *Rv1021* | 4 | *Rv2067c* | 4 | *Rv3139* | 4 |
| *Rv0172* | 1,2,3 | *Rv1022* | 9 | *Rv2068c* | 1,2,3 | *Rv3150* | 2 |
| *Rv0173* | 1,3 | *Rv1023* | 2 | *Rv2072c* | 4 | *Rv3151* | 2,4 |
| *Rv0174* | 1,2,3 | *Rv1035c* | 4 | *Rv2080* | 1,2,3 | *Rv3158* | 3 |
| *Rv0175* | 1 | *Rv1037c* | 4 | *Rv2090* | 4 | *Rv3161c* | 2 |
| *Rv0178* | 1,9 | *Rv1038c* | 4 | *Rv2092c* | 4 | *Rv3164c* | 4 |
| *Rv0183* | 4 | *Rv1041c* | 4 | *Rv2097c* | 4 | *Rv3169* | 2 |
| *Rv0187* | 2 | *Rv1050* | 3 | *Rv2101* | 4 | *Rv3190c* | 4 |
| *Rv0189c* | 2 | *Rv1051c* | 4 | *Rv2109c* | 4 | *Rv3193c* | 3 |
| *Rv0194* | 4 | *Rv1070c* | 2 | *Rv2110c* | 2 | *Rv3194c* | 3 |
| *Rv0198c* | 2,4 | *Rv1071c* | 2 | *Rv2113* | 1 | *Rv3197* | 4 |
| *Rv0199* | 1 | *Rv1073* | 4 | *Rv2124c* | 4 | *Rv3201c* | 3 |
| *Rv0200* | 9 | *Rv1074c* | 2 | *Rv2127* | 1 | *Rv3207c* | 2 |
| *Rv0202c* | 4 | *Rv1075c* | 3 | *Rv2138* | 2 | *Rv3208A* | 2 |
| *Rv0203* | 2,3,10 | *Rv1077* | 2 | *Rv2140c* | 2 | *Rv3209* | 1 |
| *Rv0204c* | 4 | *Rv1078* | 1 | *Rv2145c* | 4 | *Rv3210c* | 2 |
| *Rv0206c* | 4,7 | *Rv1079* | 2 | *Rv2149c* | 4 | *Rv3212* | 2 |
| *Rv0211* | 2 | *Rv1080c* | 2 | *Rv2158c* | 2 | *Rv3213* | 4 |
| *Rv0216* | 2 | *Rv1084* | 2 | *Rv2159c* | 4 | *Rv3214* | 2 |
| *Rv0219* | 3 | *Rv1093* | 2 | *Rv2163c* | 7 | *Rv3224* | 2 |
| *Rv0222* | 2 | *Rv1096* | 1,2 | *Rv2171* | 2 | *Rv3227* | 2 |
| *Rv0229c* | 2 | *Rv1097c* | 2 | *Rv2190* | 3 | *Rv3244c* | 2,3 |
| *Rv0231* | 2 | *Rv1098c* | 2 | *Rv2191* | 4 | *Rv3246c* | 2 |
| *Rv0234c* | 2 | *Rv1108c* | 4 | *Rv2195* | 2,4 | *Rv3248c* | 2 |
| *Rv0237* | 2,3,4 | *Rv1122* | 2 | *Rv2198c* | 2 | *Rv3253c* | 1 |
| *Rv0242c* | 2,4 | *Rv1124* | 4 | *Rv2200c* | 1,3 | *Rv3254* | 4 |
| *Rv0246* | 4 | *Rv1133c* | 2,4 | *Rv2201* | 3 | *Rv3255c* | 2 |
| *Rv0248c* | 2,4 | *Rv1138c* | 4 | *Rv2202c* | 2 | *Rv3267* | 1,2,3 |
| *Rv0260c* | 4 | *Rv1143* | 2 | *Rv2203* | 1 | *Rv3270* | 7 |
| *Rv0265c* | 1,2,3 | *Rv1157c* | 1 | *Rv2204c* | 2 | *Rv3274c* | 2 |
| *Rv0272c* | 2 | *Rv1158c* | 3 | *Rv2210c* | 2 | *Rv3276c* | 2 |
| *Rv0274* | 2 | *Rv1159A* | 2 | *Rv2211c* | 2 | *Rv3280* | 2 |
| *Rv0281* | 2 | *Rv1164* | 1 | *Rv2212* | 2 | *Rv3282* | 2 |
| *Rv0282* | 4 | *Rv1166* | 2,3 | *Rv2220* | 2,22 | *Rv3283* | 4 |
| *Rv0283* | 4 | *Rv1168c* | 14 | *Rv2223c* | 2 | *Rv3285* | 2 |
| *Rv0284* | 4 | *Rv1174c* | 1,2,3,4,15 | *Rv2224c* | 2,3 | *Rv3296* | 4 |
| *Rv0285* | 2,3 | *Rv1176c* | 4 | *Rv2229c* | 4 | *Rv3303c* | 2 |
| *Rv0287* | 2,11 | *Rv1179c* | 4 | *Rv2234* | 8 | *Rv3304* | 2 |
| *Rv0288* | 11 | *Rv1181* | 4 | *Rv2235* | 4 | *Rv3310* | 2,3,8 |
| *Rv0291* | 2,3 | *Rv1183* | 3,4 | *Rv2238c* | 2 | *Rv3312A* | 30 |
| *Rv0295c* | 4 | *Rv1186c* | 4 | *Rv2240c* | 1,2 | *Rv3318* | 2 |
| *Rv0296c* | 2,4 | *Rv1188* | 4 | *Rv2241* | 2 | *Rv3330* | 2 |
| *Rv0305c* | 4 | *Rv1198* | 2,4 | *Rv2244* | 2,4 | *Rv3336c* | 2 |
| *Rv0306* | 4 | *Rv1201c* | 2 | *Rv2245* | 2,4 | *Rv3339c* | 2 |
| *Rv0309* | 2,3 | *Rv1223* | 2 | *Rv2246* | 2,4 | *Rv3354* | 3 |
| *Rv0311* | 2 | *Rv1228* | 2 | *Rv2249c* | 4 | *Rv3356c* | 2 |
| *Rv0312* | 1 | *Rv1230c* | 1 | *Rv2251* | 2,3 | *Rv3369* | 2 |
| *Rv0315* | 2,3 | *Rv1231c* | 4 | *Rv2253* | 2,3 | *Rv3370* | 4 |
| *Rv0316* | 4 | *Rv1235* | 2 | *Rv2258c* | 2 | *Rv3372* | 2 |
| *Rv0321* | 2 | *Rv1239c* | 1 | *Rv2264c* | 1 | *Rv3389c* | 2 |
| *Rv0331* | 2 | *Rv1245c* | 4 | *Rv2280* | 2 | *Rv3390* | 1 |
| *Rv0335c* | 4 | *Rv1252c* | 2,3 | *Rv2284* | 1 | *Rv3394c* | 4 |
| *Rv0336* | 4 | *Rv1257c* | 2 | *Rv2288* | 4 | *Rv3395A* | 2 |
| *Rv0338c* | 4 | *Rv1268c* | 16 | *Rv2289* | 7 | *Rv3402c* | 3 |
| *Rv0340* | 2 | *Rv1269c* | 2,3 | *Rv2290* | 1,2 | *Rv3413c* | 1,2 |
| *Rv0342* | 4 | *Rv1270c* | 2,3 | *Rv2296* | 4 | *Rv3417c* | 4 |
| *Rv0346c* | 1 | *Rv1275* | 2 | *Rv2298* | 4 | *Rv3418c* | 2,4 |
| *Rv0350* | 2,4 | *Rv1279* | 4 | *Rv2301* | 1,2,3,18 | *Rv3423c* | 2 |
| *Rv0352* | 4 | *Rv1280c* | 9 | *Rv2314c* | 2 | *Rv3430c* | 4 |
| *Rv0357c* | 2 | *Rv1289* | 4 | *Rv2320c* | 1 | *Rv3451* | 2 |
| *Rv0361* | 1,9 | *Rv1292* | 2 | *Rv2339* | 1,4 | *Rv3455c* | 4 |
| *Rv0363c* | 2 | *Rv1293* | 4 | *Rv2344c* | 2 | *Rv3456c* | 4 |
| *Rv0383c* | 4 | *Rv1295* | 2 | *Rv2345* | 2 | *Rv3457c* | 2 |
| *Rv0386* | 4 | *Rv1297* | 4 | *Rv2346c* | 2,4 | *Rv3463* | 2 |
| *Rv0391* | 2 | *Rv1308* | 4 | *Rv2347c* | 2 | *Rv3464* | 4 |
| *Rv0398c* | 2,3 | *Rv1309* | 4 | *Rv2362c* | 4 | *Rv3465* | 2 |
| *Rv0402c* | 1,3 | *Rv1310* | 4 | *Rv2376c* | 2,3 | *Rv3476c* | 1 |
| *Rv0403c* | 2,9 | *Rv1313c* | 4 | *Rv2379* | 4 | *Rv3484* | 1,2,3 |
| *Rv0408* | 2 | *Rv1314c* | 2 | *Rv2389c* | 13 | *Rv3485c* | 3 |
| *Rv0411c* | 2,3 | *Rv1317c* | 4 | *Rv2391* | 2 | *Rv3490* | 4 |
| *Rv0412c* | 1 | *Rv1319c* | 1 | *Rv2394* | 1,2 | *Rv3491* | 2,3 |
| *Rv0418* | 1,9 | *Rv1323* | 2 | *Rv2400c* | 2 | *Rv3494c* | 1 |
| *Rv0423c* | 2 | *Rv1326c* | 2 | *Rv2404c* | 4 | *Rv3495c* | 3,4 |
| *Rv0431* | 2 | *Rv1327c* | 2 | *Rv2416c* | 23 | *Rv3496c* | 1 |
| *Rv0432* | 1 | *Rv1328* | 2 | *Rv2427c* | 2 | *Rv3497c* | 1 |
| *Rv0435c* | 4 | *Rv1340* | 2 | *Rv2429* | 2 | *Rv3498c* | 1 |
| *Rv0436c* | 3 | *Rv1352* | 2,3 | *Rv2430c* | 2,24 | *Rv3499c* | 31 |
| *Rv0440* | 2,4 | *Rv1368* | 1,2 | *Rv2431c* | 2 | *Rv3509c* | 2 |
| *Rv0445c* | 2 | *Rv1377c* | 2 | *Rv2438c* | 2,4 | *Rv3518c* | 2,4 |
| *Rv0446c* | 3 | *Rv1378c* | 4 | *Rv2441c* | 4 | *Rv3520c* | 2 |
| *Rv0450c* | 1,4,7 | *Rv1380* | 2,4 | *Rv2443* | 1 | *Rv3525c* | 2 |
| *Rv0453* | 1,3 | *Rv1382* | 3 | *Rv2445c* | 2,24 | *Rv3534c* | 2 |
| *Rv0455c* | 2,3 | *Rv1386* | 3 | *Rv2448c* | 2,4 | *Rv3545c* | 2 |
| *Rv0457c* | 2 | *Rv1388* | 4 | *Rv2450c* | 3,13 | *Rv3546* | 2 |
| *Rv0458* | 2 | *Rv1392* | 4 | *Rv2454c* | 4 | *Rv3556c* | 2 |
| *Rv0462* | 2 | *Rv1407* | 4 | *Rv2457c* | 4 | *Rv3568c* | 2 |
| *Rv0464c* | 4 | *Rv1411c* | 2 | *Rv2461c* | 2 | *Rv3569c* | 4 |
| *Rv0467* | 2 | *Rv1412* | 2 | *Rv2465c* | 2 | *Rv3572* | 2,3 |
| *Rv0468* | 2 | *Rv1419* | 1,2,3 | *Rv2467* | 2 | *Rv3584* | 1,2,3 |
| *Rv0475* | 4 | *Rv1420* | 4 | *Rv2469c* | 3 | *Rv3587c* | 2,3 |
| *Rv0477* | 2,3 | *Rv1423* | 4 | *Rv2476c* | 2,4 | *Rv3597c* | 4 |
| *Rv0480c* | 4 | *Rv1435c* | 1,2,3 | *Rv2477c* | 2 | *Rv3607c* | 2 |
| *Rv0483* | 1,2 | *Rv1436* | 2 | *Rv2503c* | 2 | *Rv3614c* | 32 |
| *Rv0489* | 2 | *Rv1437* | 2 | *Rv2506* | 2 | *Rv3615c* | 33 |
| *Rv0490* | 3 | *Rv1448c* | 2 | *Rv2516c* | 4 | *Rv3616c* | 34 |
| *Rv0500* | 2 | *Rv1449c* | 2 | *Rv2524c* | 4 | *Rv3624c* | 2 |
| *Rv0503c* | 2 | *Rv1453* | 4 | *Rv2525c* | 2 | *Rv3627c* | 2,3 |
| *Rv0506* | 1,2,3 | *Rv1454c* | 2,4 | *Rv2537c* | 2 | *Rv3629c* | 3 |
| *Rv0507* | 2,4 | *Rv1464* | 2 | *Rv2538c* | 4 | *Rv3630* | 9 |
| *Rv0510* | 2 | *Rv1467c* | 4 | *Rv2540c* | 2 | *Rv3646c* | 4 |
| *Rv0512* | 2 | *Rv1474c* | 4 | *Rv2544* | 3 | *Rv3648c* | 4 |
| *Rv0517* | 1 | *Rv1475c* | 2,4 | *Rv2553c* | 4 | *Rv3654c* | 35 |
| *Rv0518* | 2 | *Rv1477* | 2,3 | *Rv2557* | 2 | *Rv3655c* | 35 |
| *Rv0519c* | 2 | *Rv1478* | 4 | *Rv2558* | 2 | *Rv3666c* | 2 |
| *Rv0524* | 2 | *Rv1479* | 4 | *Rv2563* | 3 | *Rv3667* | 4 |
| *Rv0526* | 2,3 | *Rv1485* | 2 | *Rv2572c* | 4 | *Rv3668c* | 2,3 |
| *Rv0546c* | 2 | *Rv1488* | 3 | *Rv2575* | 2 | *Rv3671c* | 2,3 |
| *Rv0549c* | 2 | *Rv1492* | 2,4 | *Rv2576c* | 2,3 | *Rv3674c* | 4 |
| *Rv0555* | 2 | *Rv1493* | 2 | *Rv2579* | 2 | *Rv3678c* | 2 |
| *Rv0556* | 9 | *Rv1498A* | 2 | *Rv2580c* | 4 | *Rv3681c* | 4 |
| *Rv0559c* | 2,3 | *Rv1503c* | 4 | *Rv2583c* | 4 | *Rv3682* | 2,3 |
| *Rv0561c* | 4 | *Rv1511* | 2 | *Rv2585c* | 1,3 | *Rv3684* | 2 |
| *Rv0563* | 3 | *Rv1515c* | 4 | *Rv2586c* | 4 | *Rv3688c* | 2 |
| *Rv0571c* | 4 | *Rv1520* | 4 | *Rv2587c* | 7 | *Rv3689* | 1 |
| *Rv0577* | 2,12 | *Rv1531* | 2 | *Rv2589* | 2 | *Rv3693* | 3 |
| *Rv0580c* | 4 | *Rv1536* | 2,4 | *Rv2599* | 1 | *Rv3696c* | 2 |
| *Rv0583c* | 1,2,3 | *Rv1541c* | 3 | *Rv2601* | 4 | *Rv3705c* | 2,3 |
| *Rv0594* | 1 | *Rv1550* | 4 | *Rv2602* | 2 | *Rv3710* | 2 |
| *Rv0605* | 4 | *Rv1564c* | 2 | *Rv2607* | 2 | *Rv3717* | 2 |
| *Rv0613c* | 4 | *Rv1566c* | 7 | *Rv2609c* | 4 | *Rv3722c* | 2 |
| *Rv0629c* | 4 | *Rv1567c* | 1 | *Rv2623* | 4 | *Rv3725* | 3 |
| *Rv0630c* | 4 | *Rv1568* | 2 | *Rv2639c* | 1 | *Rv3741c* | 4 |
| *Rv0631c* | 4 | *Rv1591* | 1 | *Rv2650c* | 4 | *Rv3755c* | 2 |
| *Rv0632c* | 2 | *Rv1599* | 2 | *Rv2668* | 2,3 | *Rv3759c* | 2,3 |
| *Rv0636* | 2,4 | *Rv1600* | 4 | *Rv2672* | 2,3 | *Rv3760* | 3 |
| *Rv0640* | 4 | *Rv1612* | 2 | *Rv2676c* | 2 | *Rv3763* | 2 |
| *Rv0642c* | 4 | *Rv1617* | 2 | *Rv2679* | 2 | *Rv3772* | 2 |
| *Rv0645c* | 2 | *Rv1620c* | 4 | *Rv2691* | 2 | *Rv3774* | 2 |
| *Rv0647c* | 4 | *Rv1627c* | 2 | *Rv2693c* | 3 | *Rv3779* | 1 |
| *Rv0652* | 4 | *Rv1630* | 2,4 | *Rv2697c* | 2 | *Rv3786c* | 2,4 |
| *Rv0655* | 4 | *Rv1635c* | 1 | *Rv2714* | 2 | *Rv3794* | 7 |
| *Rv0667* | 2,4 | *Rv1637c* | 2 | *Rv2716* | 2 | *Rv3795* | 4 |
| *Rv0668* | 2 | *Rv1638* | 4 | *Rv2721c* | 1,2,3 | *Rv3796* | 2 |
| *Rv0669c* | 4 | *Rv1648* | 1 | *Rv2731* | 4 | *Rv3797* | 2 |
| *Rv0672* | 2,4 | *Rv1650* | 4 | *Rv2748c* | 4,7 | *Rv3800c* | 2,4 |
| *Rv0673* | 4 | *Rv1652* | 2 | *Rv2752c* | 2 | *Rv3801c* | 4 |
| *Rv0674* | 3 | *Rv1655* | 2 | *Rv2766c* | 3 | *Rv3802c* | 1,2 |
| *Rv0676c* | 1,7 | *Rv1656* | 2 | *Rv2780* | 2 | *Rv3803c* | 2,3 |
| *Rv0677c* | 1,2,3 | *Rv1665* | 2 | *Rv2783c* | 2 | *Rv3804c* | 2,3,4,7 |
| *Rv0680c* | 2,3 | *Rv1691* | 4 | *Rv2787* | 4 | *Rv3825c* | 4 |
| *Rv0684* | 2 | *Rv1696* | 4 | *Rv2790c* | 2,4 | *Rv3835* | 1,2,3 |
| *Rv0685* | 4 | *Rv1700* | 2 | *Rv2791c* | 4 | *Rv3841* | 2 |
| *Rv0706* | 4 | *Rv1703c* | 2,4 | *Rv2793c* | 4 | *Rv3846* | 2,36 |
| *Rv0711* | 2 | *Rv1707* | 1 | *Rv2799* | 2,3 | *Rv3849* | 4,37 |
| *Rv0715* | 4 | *Rv1713* | 4 | *Rv2800* | 4 | *Rv3853* | 2 |
| *Rv0719* | 4 | *Rv1728c* | 1 | *Rv2812* | 4 | *Rv3854c* | 4 |
| *Rv0725c* | 4 | *Rv1742* | 4 | *Rv2817c* | 4 | *Rv3859c* | 2 |
| *Rv0731c* | 2 | *Rv1743* | 1 | *Rv2831* | 2 | *Rv3863* | 4 |
| *Rv0732* | 3 | *Rv1746* | 2 | *Rv2839c* | 4 | *Rv3865* | 34 |
| *Rv0733* | 2 | *Rv1747* | 4 | *Rv2844* | 2 | *Rv3869* | 1,2 |
| *Rv0753c* | 2 | *Rv1759c* | 3 | *Rv2845c* | 2,4 | *Rv3872* | 2 |
| *Rv0761c* | 2 | *Rv1771* | 4 | *Rv2847c* | 4 | *Rv3873* | 38 |
| *Rv0762c* | 4 | *Rv1774* | 2 | *Rv2848c* | 3 | *Rv3874* | 2,4,34 |
| *Rv0772* | 2 | *Rv1779c* | 1 | *Rv2854* | 4 | *Rv3875* | 2,34 |
| *Rv0774c* | 2,3 | *Rv1782* | 2 | *Rv2855* | 2 | *Rv3881c* | 2,39 |
| *Rv0775* | 4 | *Rv1794* | 2 | *Rv2858c* | 2 | *Rv3883c* | 2,40 |
| *Rv0777* | 2,4 | *Rv1804c* | 3 | *Rv2860c* | 2 | *Rv3899c* | 3 |
| *Rv0783c* | 1 | *Rv1810* | 2,3 | *Rv2861c* | 2 | *Rv3901c* | 1 |
| *Rv0787* | 2,3 | *Rv1812c* | 3 | *Rv2869c* | 2 | *Rv3903c* | 41 |
| *Rv0794c* | 2 | *Rv1815* | 2,3 | *Rv2873* | 2,3,26 | *Rv3907c* | 4 |
| *Rv0799c* | 2 | *Rv1817* | 2,4 | *Rv2874* | 3 | *Rv3910* | 1 |
| *Rv0800* | 2 | *Rv1818c* | 17 | *Rv2875* | 2,3 | *Rv3914* | 2 |
| *Rv0806c* | 4 | *Rv1819c* | 1 | *Rv2878c* | 2,3,27 | *Rv3917c* | 3 |
| *Rv0811c* | 2 | *Rv1825* | 2 | *Rv2882c* | 2 | *Rv2935* | 4 |
| *Rv0815c* | 4 | *Rv1827* | 2,18 | *Rv2889c* | 2 | *Rv2938* | 1 |
| *Rv0821c* | 2 | *Rv1832* | 2,4 | *Rv2903c* | 7 | *Rv2940c* | 2,4 |
| *Rv0822c* | 2 | *Rv1836c* | 1,2 | *Rv2905* | 1,3 | *Rv2941* | 2 |
| *Rv0824c* | 4 | *Rv1837c* | 2 | *Rv2911* | 2,3 | *Rv2945c* | 1,2,3,4 |
| *Rv0831c* | 2,4 | *Rv1843c* | 2 | *Rv2916c* | 4 | *Rv2967c* | 4 |

REFERENCES

1. Genome-wide identification of Mycobacterium tuberculosis exported proteins with roles in intracellular growth. McCann JR, McDonough JA, Sullivan JT, Feltcher ME, Braunstein M. J Bacteriol. 2011 Feb;193(4):854-61. doi: 10.1128/JB.01271-10. Epub 2010 Dec 10.
2. Bacterial proteins with cleaved or uncleaved signal peptides of the general secretory pathway. de Souza GA, Leversen NA, Malen H, Wiker HG. J Proteomics (2011) 75(2):502-10.
3. Comprehensive analysis of exported proteins from Mycobacterium tuberculosis H37*Rv*. Malen H, Be*Rv*en FS, Fladmark KE, Wiker HG. Proteomics (2007) 7(10):1702-18.
4. Mycobacterium tuberculosis functional network analysis by global subcellular protein profiling. Mawuenyega KG, Forst CV, Dobos KM, Belisle JT, Chen J, Bradbury EM, Bradbury AR, Chen X. Mol Biol Cell (2005) 16(1):396-404
5. Mycobacterium tuberculosis cyclophilin A uses novel signal sequence for secretion and mimics eukaryotic cyclophilins for interaction with host protein repertoire. Bhaduri A, Misra R, Maji A, Bhetaria PJ, Mishra S, Arora G, Singh LK, Dhasmana N, Dubey N, Virdi JS, Singh Y. PLoS One. 2014 Feb 4;9(2):e88090. PMID: 24505389; Two-dimensional electrophoresis for analysis of Mycobacterium tuberculosis culture filtrate and purification and characterization of six novel proteins. Weldingh K1, Rosenkrands I, Jacobsen S, Rasmussen PB, Elhay MJ, Andersen P. Infect Immun. 1998 Aug;66(8):3492-500.
6. MTC28, a novel 28-kilodalton proline-rich secreted antigen specific for the Mycobacterium tuberculosis complex. Manca C, Lyashchenko K, Colangeli R, Gennaro ML. Infect Immun. 1997 Dec;65(12):4951-7. PMID: 9393781
7. Identification of genes encoding exported Mycobacterium tuberculosis proteins using a Tn552'phoA in vitro transposition system. Braunstein M, Griffin TJ IV, Kriakov JI, Friedman ST, Grindley ND, Jacobs WR Jr. J Bacteriol. 2000 May;182(10):2732-40. PMID: 10781540
8. Mycobacterium tuberculosis-secreted phosphatases: from pathogenesis to targets for TB drug development. Wong D, Chao JD, Av-Gay Y. Trends Microbiol. 2013 Feb;21(2):100-9. Review. PMID: 23084287
9. Computational prediction and experimental assessment of secreted/surface proteins from Mycobacterium tuberculosis H37*Rv*. Vizcaíno C, Restrepo-Montoya D, Rodríguez D, Niño LF, Ocampo M, Vanegas M, Reguero MT, Martínez NL, Patarroyo ME, Patarroyo MA. PLoS Comput Biol. 2010 Jun 24;6(6):e1000824. PMID: 20585611
10. The Mycobacterium tuberculosis secreted protein *Rv*0203 transfers heme to membrane proteins MmpL3 and MmpL11. Owens CP, Chim N, Graves AB, Harmston CA, Iniguez A, Contreras H, Liptak MD, Goulding CW. J Biol Chem. 2013 Jul 26;288(30):21714-28. PMID: 23760277
11. Mycobacterium tuberculosis type VII secreted effector EsxH targets host ESCRT to impair trafficking. Mehra A, Zahra A, Thompson V, Sirisaengtaksin N, Wells A, Porto M, Köster S, Penberthy K, Kubota Y, Dricot A, Rogan D, Vidal M, Hill DE, Bean AJ, Philips JA. PLoS Pathog. 2013 Oct;9(10):e1003734. PMID: 24204276
12. The Mycobacterium tuberculosis complex-restricted gene cfp32 encodes an expressed protein that is detectable in tuberculosis patients and is positively correlated with pulmonary interleukin-10. Huard RC, Chitale S, Leung M, Lazzarini LC, Zhu H, Shashkina E, Laal S, Conde MB, Kritski AL, Belisle JT, Kreiswirth BN, Lapa e Silva JR, Ho JL. Infect Immun. 2003 Dec;71(12):6871-83. PMID: 14638775
13. The resuscitation-promoting factors of Mycobacterium tuberculosis are required for virulence and resuscitation from dormancy but are collectively dispensable for growth in vitro. Kana BD, Gordhan BG, Downing KJ, Sung N, Vostroktunova G, Machowski EE, Tsenova L, Young M, Kaprelyants A, Kaplan G, Mizrahi V. Mol Microbiol. 2008 Feb;67(3):672-84. PMID: 18186793
14. The PPE domain of PPE17 is responsible for its surface localization and can be used to express heterologous proteins on the mycobacterial surface. Donà V, Ventura M, Sali M, Cascioferro A, Provvedi R, Palù G, Delogu G, Manganelli R. PLoS One. 2013;8(3):e57517. PMID: 23469198
15. Molecular cloning and immunologic reactivity of a novel low molecular mass antigen of Mycobacterium tuberculosis. Coler RN, Skeiky YA, Vedvick T, Bement T, Ovendale P, Campos-Neto A, Alderson MR, Reed SG. J Immunol. 1998 Sep 1;161(5):2356-64. PMID: 9725231
16. *Rv*1268c protein peptide inhibiting Mycobacterium tuberculosis H37*Rv* entry to target cells. Ocampo M1, Rodríguez DC, Rodríguez J, Bermúdez M, Muñoz CM, Patarroyo MA, Patarroyo ME. Bioorg Med Chem. 2013 Nov 1;21(21):6650-6. PMID: 23993672
17. Functional dissection of the PE domain responsible for translocation of PE_PGRS33 across the mycobacterial cell wall. Cascioferro A, Daleke MH, Ventura M, Donà V, Delogu G, Palù G, Bitter W, Manganelli R. PLoS One. 2011;6(11):e27713. PMID: 22110736
18. Two-dimensional electrophoresis for analysis of Mycobacterium tuberculosis culture filtrate and purification and characterization of six novel proteins. Weldingh K1, Rosenkrands I, Jacobsen S, Rasmussen PB, Elhay MJ, Andersen P. Infect Immun. 1998 Aug;66(8):3492-500.
19. Distinct differences in repertoires of low-molecular-mass secreted antigens of Mycobacterium avium complex and Mycobacterium tuberculosis. Olsen I, Reitan LJ, Wiker HG. J Clin Microbiol. 2000 Dec;38(12):4453-8. PMID: 11101579
20. Characterization of the secreted chorismate mutase from the pathogen Mycobacterium tuberculosis. Sasso S, Ramakrishnan C, Gamper M, Hilvert D, Kast P. FEBS J. 2005 Jan;272(2):375-89. PMID: 15654876
21. Guinea pig cellular immune responses to proteins secreted by Mycobacterium tuberculosis. Hasløv K, Andersen A, Nagai S, Gottschau A, Sørensen T, Andersen P. Infect Immun. 1995 Mar;63(3):804-10. PMID: 7868250
22. High extracellular levels of Mycobacterium tuberculosis glutamine synthetase and superoxide dismutase in actively growing cultures are due to high expression and extracellular stability rather than to a protein-specific export mechanism. Tullius MV, Harth G, Horwitz MA. Infect Immun. 2001 Oct;69(10):6348-63. PMID: 11553579
23. Subcellular localization of the Iitracellular su*Rv*ival-enhancing Eis protein of Mycobacterium tuberculosis. Dahl JL1, Wei J, Moulder JW, Laal S, Friedman RL. Infect Immun. 2001 Jul;69(7):4295-302. PMID: 11401966; Eis (enhanced intracellular su*Rv*ival) protein of Mycobacterium tuberculosis disturbs the cross regulation of T-cells. Lella RK1, Sharma C. J Biol Chem. 2007 Jun 29;282(26):18671-5. PMID: 17449476
24. A specific secretion system mediates PPE41 transport in pathogenic mycobacteria. Abdallah AM, Verboom T, Hannes F, Safi M, Strong M, Eisenberg D, Musters RJ, Vandenbroucke-Grauls CM, Appelmelk BJ, Luirink J, Bitter W. Mol Microbiol. 2006 Nov;62(3):667-79. PMID: 17076665
25. Cytotoxic activity of nucleoside diphosphate kinase secreted from Mycobacterium tuberculosis. Chopra P, Singh A, Koul A, Ramachandran S, Drlica K, Tyagi AK, Singh Y. Eur J Biochem. 2003 Feb;270(4):625-34. PMID: 12581202
26. Molecular characterization of MPT83: a seroreactive antigen of Mycobacterium tuberculosis with homology to MPT70. Hewinson RG, Michell SL, Russell WP, McAdam RA, Jacobs WR (1996) Scand J Immunol 43: 490–499.
27. Characterization of the secreted MPT53 antigen of Mycobacterium tuberculosis. Johnson S, Brusasca P, Lyashchenko K, Spencer JS, Wiker HG, Bifani P, Shashkina E, Kreiswirth B, Harboe M, Schluger N, Gomez M, Gennaro ML. Infect Immun. 2001 Sep;69(9):5936-9. PMID: 11500477
28. Structure analysis of the extracellular domain reveals disulfide bond forming-protein properties of Mycobacterium tuberculosis *Rv*2969c. Wang L, Li J, Wang X, Liu W, Zhang XC, Li X, Rao Z. Protein Cell. 2013 Aug;4(8):628-40. PMID: 23828196
29. Conse*Rv*ed Pro-Glu (PE) and Pro-Pro-Glu (PPE) protein domains target LipY lipases of pathogenic mycobacteria to the cell surface via the ESX-5 pathway. Daleke MH, Cascioferro A, de Punder K, Ummels R, Abdallah AM, van der Wel N, Peters PJ, Luirink J, Manganelli R, Bitter W. J Biol Chem. 2011 May 27;286(21):19024-34. PMID: 21471225
30. Mycobacterium tuberculosis produces pili during human infection. Alteri CJ1, Xicohténcatl-Cortes J, Hess S, Caballero-Olín G, Girón JA, Friedman RL. Proc Natl Acad Sci U S A. 2007 Mar 20;104(12):5145-50. PMID: 17360408
31. Characterization of Mce4A protein of Mycobacterium tuberculosis: role in invasion and su*Rv*ival. Saini NK1, Sharma M, Chandolia A, Pasricha R, Brahmachari V, Bose M. BMC Microbiol. 2008 Nov 19;8:200. PMID: 19019220
32. EspD is critical for the virulence-mediating ESX-1 secretion system in Mycobacterium tuberculosis. Chen JM1, Boy-Röttger S, Dhar N, Sweeney N, Buxton RS, Pojer F, Rosenkrands I, Cole ST. J Bacteriol. 2012 Feb;194(4):884-93. PMID: 22155774
33. *Rv*3615c is a highly immunodominant RD1 (Region of Difference 1)-dependent secreted antigen specific for Mycobacterium tuberculosis infection. Millington KA, Fortune SM, Low J, Garces A, Hingley-Wilson SM, Wickremasinghe M, Kon OM, Lalvani A. Proc Natl Acad Sci U S A. 2011 Apr 5;108(14):5730-5. PMID: 21427227; ESX-1 secreted virulence factors are recognized by multiple cytosolic AAA ATPases in pathogenic mycobacteria. Champion PA, Champion MM, Manzanillo P, Cox JS. Mol Microbiol. 2009 Sep;73(5):950-62. PMID: 19682254
34. ESX-1 secreted virulence factors are recognized by multiple cytosolic AAA ATPases in pathogenic mycobacteria. Champion PA, Champion MM, Manzanillo P, Cox JS. Mol Microbiol. 2009 Sep;73(5):950-62. PMID: 19682254; Mutually dependent secretion of proteins required for mycobacterial virulence. Fortune SM, Jaeger A, Sarracino DA, Chase MR, Sassetti CM, Sherman DR, Bloom BR, Rubin EJ. Proc Natl Acad Sci U S A. 2005 Jul 26;102(30):10676-81. PMID: 16030141
35. Secreted Mycobacterium tuberculosis *Rv*3654c and *Rv*3655c proteins participate in the suppression of macrophage apoptosis. Danelishvili L, Yamazaki Y, Selker J, Bermudez LE. PLoS One. 2010 May 4;5(5):e10474. PMID: 20454556
36. SecA2 functions in the secretion of superoxide dismutase A and in the virulence of Mycobacterium tuberculosis. Braunstein M, Espinosa BJ, Chan J, Belisle JT, Jacobs WR Jr. Mol Microbiol. 2003 Apr;48(2):453-64. PMID: 12675804; High extracellular levels of Mycobacterium tuberculosis glutamine synthetase and superoxide dismutase in actively growing cultures are due to high expression and extracellular stability rather than to a protein-specific export mechanism. Tullius MV, Harth G, Horwitz MA. Infect Immun. 2001 Oct;69(10):6348-63. PMID: 11553579
37. Secreted transcription factor controls Mycobacterium tuberculosis virulence. Raghavan S1, Manzanillo P, Chan K, Dovey C, Cox JS. Nature. 2008 Aug 7;454(7205):717-21. PMID: 18685700
38. Serodiagnosis efficacy and immunogenicity of the fusion protein of Mycobacterium tuberculosis composed of the 10-kilodalton culture filtrate protein, ESAT-6, and the extracellular domain fragment of PPE68. Xu JN, Chen JP, Chen DL. Clin Vaccine Immunol. 2012 Apr;19(4):536-44. doi: 10.1128/CVI.05708-11. Epub 2012 Feb 22. PMID: 22357648
39. A mycobacterium ESX-1-secreted virulence factor with unique requirements for export. McLaughlin B, Chon JS, MacGurn JA, Carlsson F, Cheng TL, Cox JS, Brown EJ. PLoS Pathog. 2007 Aug 3;3(8):e105. PMID: 17676952; ESX-1 secreted virulence factors are recognized by multiple cytosolic AAA ATPases in pathogenic mycobacteria. Champion PA, Champion MM, Manzanillo P, Cox JS. Mol Microbiol. 2009 Sep;73(5):950-62. PMID: 19682254
40. Mycosin-1, a subtilisin-like serine protease of Mycobacterium tuberculosis, is cell wall-associated and expressed during infection of macrophages. Dave JA, Gey van Pittius NC, Beyers AD, Ehlers MR, Brown GD. BMC Microbiol. 2002 Oct 7;2:30. PMID: 12366866
41. An outer membrane channel protein of Mycobacterium tuberculosis with exotoxin activity. Danilchanka O, Sun J, Pavlenok M, Maueröder C, Speer A, Siroy A, Marrero J, Trujillo C, Mayhew DL, Doornbos KS, Muñoz LE, Herrmann M, Ehrt S, Berens C, Niederweis M. Proc Natl Acad Sci U S A. 2014 May 6;111(18):6750-5. Epub 2014 Apr 21. PMID: 24753609
