# Supplementary material for: Comprehensive definition of human immunodominant CD8 antigens in tuberculosis
Source: NPJ Vaccines. 2017 Apr 3;2:8. doi: 10.1038/s41541-017-0008-6 (PMC5538316; doi:10.1038/s41541-017-0008-6)
Supplement: Supplementary file 7 — Supplementary Table S6 [file 41541_2017_8_MOESM7_ESM.docx]

**Table S6. “No response” peptide pools**

| Rv Number  (# peptides in pool) | Name |
| --- | --- |
| *Rv0040c*(25) : *Rv0173*(25) | *mtc28* : *lprK* |
| *Rv0173*(50) | *lprK* |
| *Rv0178*(30) : *Rv0173*(20) | *Rv0178* : *lprK* |
| *Rv0178*(29) : *Rv0192A*(21) | *Rv0178* : *Rv0192A* |
| *Rv0593*(42) : *Rv0584*(8) | *lprL* : *Rv0584* |
| *Rv0870c*(27) : *Rv0867c*(23) | *Rv0870c* : *rpfA* |
| *Rv1970*(38) : *Rv1860*(12) | *lprM* : *apa* |
| *Rv2253*(39) : *Rv2376c*(7) : *Rv1970*(4) | *Rv2253* : *cfp2* : *lprM* |
| *Rv2376c*(33) : *Rv2389c*(17) | *cfp2* : *rpfD* |
| *Rv2721c*(50) | *Rv2721c* |
| *Rv2721c*(50) | *Rv2721c* |
| *Rv2875*(37) : *Rv2873*(13) | *mpt70* : *mpt83* |
| *Rv2226*(50) | *Rv2226* |
| *Rv2226*(50) | *Rv2226* |
| *Rv3254*(50) | *Rv3254* |
| *Rv3717*(42) : *Rv3811*(8) | *Rv3717* : *Rv3811* |
| *Rv3811*(50) | *Rv3811* |
| *Rv3896c*(47) : *Rv3909*(3) | *Rv3896c* : *Rv3909* |
| *Rv0590*(41) : *Rv0589*(9) | *mce2B* : *mce2A* |
| *Rv3496c*(50) | *mce4D* |
| *Rv0102*(41) : *Rv3499c*(9) | *Rv0102* : *mce4A* |
| *Rv1733c*(39) : *Rv1730c*(11) | *Rv1733c* : *Rv1730c* |
| *Rv2834c*(43) : *Rv2833c*(7) | *ugpE* : *ugpB* |
| *Rv2835c*(46) : *Rv2877c*(4) | *ugpA* : *Rv2877c* |
| *Rv0456c*(50) | *echA2* |
| *Rv0109*(26) : *Rv0096*(24) | *PE_PGRS1* : *PPE1* |
| *Rv0297*(50) | *PE_PGRS5* |
| *Rv0304c*(50) | *PPE5* |
| *Rv0872c*(50) | *PE_PGRS15* |
| *Rv1386*(23) : *Rv1387*(20) : *Rv1361c*(7) | *PE15* : *PPE20* : *PPE19* |
| *Rv1450c*(46) : *Rv1441c*(4) | *PE_PGRS27* : *PE_PGRS26* |
| *Rv1450c*(50) | *PE_PGRS27* |
| *Rv1753c*(30) | *PPE24* |
| *Rv1802*(50) | *PPE30* |
| *Rv2769c*(37) : *Rv2770c*(13) | *PE27* : *PPE44* |
| *Rv2770c*(30) : *Rv2853*(20) | *PPE44* : *PE_PGRS48* |
| *Rv2892c*(31) : *Rv3018c*(14) : *Rv3018A*(5) | *PPE45* : *PPE46* : *PE27A* |
| *Rv3021c*(50) | *PPE47* |
| *Rv3021c*(29) : *Rv3022A*(21) | *PPE47* : *PE29* |
| *Rv3097c*(50) | *PE_PGRS63* |
| *Rv3125c*(50) | *PPE49* |
| *Rv3159c*(42) : *Rv3343c*(8) | *PPE53* : *PPE54* |
| *Rv3343c*(50) | *PPE54* |
| *Rv3347c*(50) | *PPE55* |
| *Rv3350c*(50) | *PPE56* |
| *Rv3367*(50) | *PE_PGRS51* |
| *Rv3388*(50) | *PE_PGRS52* |
| *Rv3429*(42) : *Rv3426*(8) | *PPE59* : *PPE58* |
| *Rv3478*(50) | *PPE60* |
| *Rv3507*(50) | *PE_PGRS53* |
| *Rv3507*(50) | *PE_PGRS53* |
| *Rv3507*(50) | *PE_PGRS53* |
| *Rv3508*(50) | *PE_PGRS54* |
| *Rv3508*(50) | *PE_PGRS54* |
| *Rv3558*(42) : *Rv3590c*(8) | *PPE64* : *PE_PGRS58* |
| *Rv3738c*(43) : *Rv3653*(7) | *PPE66* : *PE_PGRS61* |
| *Rv0351*(39) : *Rv0350*(11) | *grpE* : *dnaK* |
| *Rv0384c*(33) : *Rv0351*(17) | *clpB* : *grpE* |
