# Supplementary material for: Comprehensive definition of human immunodominant CD8 antigens in tuberculosis
Source: NPJ Vaccines. 2017 Apr 3;2:8. doi: 10.1038/s41541-017-0008-6 (PMC5538316; doi:10.1038/s41541-017-0008-6)
Supplement: Supplementary file 8 — Supplementary Table S7 [file 41541_2017_8_MOESM8_ESM.docx]

**Table S7^1^. Clinical validation of immunodominant peptide pools in Mtb-infected individuals in Kampala, Uganda**

|  | | **LTBI** | | | **TB disease** | | | **All MTB Infected** | | |
| --- | --- | --- | --- | --- | --- | --- | --- | --- | --- | --- |
| Rv Number  (# peptides in pool) | Name | n | # positive (% positive) | Mean of positive responses | n | # positive (% positive) | Mean of positive responses | n | # positive (% positive) | Mean of positive responses |
| *Rv1172c*(32) : *Rv1195*(18) | *PE12* : *PE13* | 13 | 8 (62) | 45.6 | 8 | 3 (38) | 148.7 | 21 | 11 (52) | 73.7 |
| *Rv0824c*(50) | *desA1* | 10 | 5 (50) | 31.6 | 14 | 6 (43) | 46.3 | 24 | 11 (46) | 39.6 |
| *Rv1243c*(50) | *PE_PGRS23* | 11 | 4 (36) | 49.5 | 7 | 5 (71) | 69.8 | 18 | 9 (50) | 60.8 |
| *Rv1788*(22): *Rv1787*(17): *Rv1789*(11) | *PE18*: *PPE25*: *PPE26* | 10 | 4 (40) | 34.8 | 14 | 4 (29) | 43.7 | 24 | 8 (33) | 39.3 |
| *Rv1038c* (28)^2^ | *EsxJ* | 10 | 3 (30) | 25.2 | 9 | 4 (44) | 79.6 | 19 | 7 (37) | 56.3 |
| *Rv3345c*(50)^3^ | *PE_PGRS50* | 11 | 3 (27) | 28.3 | 8 | 4 (50) | 68.6 | 19 | 7 (37) | 51.3 |
| *Rv3345c*(50)^3^ | *PE_PGRS50* | 11 | 2 (11) | 33.5 | 8 | 5 (63) | 79.3 | 19 | 7 (37) | 66.2 |
| *Rv3136*(46) : *Rv3135*(4) | *PPE51* : *PPE50* | 11 | 7 (64) | 53.6 | 11 | 0 (0) | 0.0 | 22 | 7 (32) | 53.6 |
| *Rv1039c*(50) | *PPE15* | 9 | 2 (22) | 45.7 | 9 | 4 (44) | 12.5 | 18 | 6 (33) | 23.6 |
| *Rv0159c*(50) | *PE3* | 11 | 5 (45) | 29.7 | 9 | 1 (11) | 19.2 | 20 | 6 (30) | 28.0 |
| *Rv1997*(50) | *ctpF* | 11 | 5 (45) | 27.2 | 9 | 1 (11) | 40.7 | 20 | 6 (30) | 29.5 |
| *Rv3874* | *CFP10* | 9 | 5 (56) | 33.4 | 10 | 6 (60) | 38.0 | 19 | 11 (58) | 35.9 |
| *Rv0287* | *EsxG* | 9 | 3 (33) | 33.3 | 10 | 7 (70) | 111.9 | 19 | 10 (53) | 88.3 |
| *Rv0284*(17) : *Rv0288*(11) | *Rv0284* : *esxH* | 11 | 5 (45) | 53.0 | 7 | 4 (57) | 67.7 | 18 | 9 (50) | 59.5 |
| *Rv1037c* (30)^4^ | *Mtb9.9* | 9 | 6 (67) | 32.9 | 10 | 3 (30) | 45.1 | 19 | 9 (47) | 37.0 |
| *Rv3347c*(50) | *PPE55* | 8 | 4 (50) | 109.2 | 9 | 3 (33) | 90.3 | 17 | 7 (41) | 101.1 |
| *Rv3163c*(41) : *Rv3194c*(9) | *Rv3163c* : *Rv3194c* | 8 | 3 (38) | 122.4 | 9 | 3 (33) | 75.4 | 17 | 6 (35) | 98.9 |
| *Rv1996*(50) | *Rv1996* | 11 | 3 (27) | 27.3 | 14 | 3 (21) | 13.3 | 25 | 6 (24) | 20.3 |
| *Rv2270*(32): *Rv2093c*(18) | *lppN*: *tatC* | 10 | 3 (30) | 31.7 | 16 | 2 (13) | 18.0 | 26 | 5 (19) | 26.2 |
| *Rv1969*(35): *Rv1971*(15) | *mce3D*: *mce3F* | 10 | 1 (10) | 11.0 | 11 | 3 (27) | 17.0 | 21 | 4 (19) | 15.5 |
| *Rv1983*(36): *Rv1918c*(14) | *PE_PGRS35*: *PPE35* | 11 | 2 (18) | 21.5 | 13 | 2 (15) | 13.5 | 24 | 4 (17) | 17.5 |
| *Rv3555c*(48): *Rv2557*(2) | *Rv3555c*: *Rv2557* | 11 | 3 (27) | 21.0 | 14 | 1 (7) | 11.0 | 25 | 4 (16) | 18.5 |
| *Rv2041c*(50) | *Rv2041c* | 9 | 2 (22) | 25.9 | 9 | 1 (11) | 12.2 | 18 | 3 (17) | 21.3 |
| *Rv1997*(50) | *ctpF* | 11 | 2 (18) | 19.3 | 9 | 1 (11) | 39.2 | 20 | 3 (15) | 25.9 |
| *Rv3641c*(33) | *fic* | 11 | 3 (27) | 27.5 | 11 | 0 (0) | 0.0 | 22 | 3 (14) | 27.5 |
| *Rv3514*(47) : *Rv3532*(3) | *PE_PGRS57* : *PPE61* | 11 | 3 (27) | 58.4 | 11 | 0 (0) | 0.0 | 22 | 3 (14) | 58.4 |
| *Rv2672*(38): *Rv2780*(12) | *Rv2672*: *ald* | 10 | 1 (10) | 14.0 | 12 | 2 (17) | 13.0 | 22 | 3 (14) | 13.3 |
| *Rv1992c*(40): *Rv1987*(10) | *ctpG*: *Rv1987* | 10 | 3 (30) | 14.7 | 13 | 0 (0) | 0.0 | 23 | 3 (13) | 14.7 |
| *Rv2686c*(44): *Rv2687c*(6) | *Rv2686c*: *Rv2687c* | 10 | 2 (20) | 39.0 | 15 | 1 (7) | 11.0 | 25 | 3 (12) | 29.7 |
| *Rv1966*(49): *Rv1967*(1) | *mce3A*: *mce3B* | 10 | 1 (10) | 10.0 | 12 | 1 (8) | 10.0 | 22 | 2 (9) | 10.0 |
| *Rv1132*(33): *Rv1184c*(17) | *Rv1132*: *Rv1184c* | 10 | 0 (0) | 0.0 | 12 | 2 (17) | 31.0 | 22 | 2 (9) | 31.0 |
| *Rv1992c*(50) | *ctpG* | 10 | 2 (20) | 15.0 | 14 | 0 (0) | 0.0 | 24 | 2 (8) | 15.0 |
| *Rv1706c*(50) | *PPE23* | 9 | 1 (11) | 40.7 | 9 | 1 (11) | 12.3 | 18 | 2 (11) | 26.5 |
| *Rv2041c*(43) : *Rv2093c*(7) | *Rv2041c* : *tatC* | 9 | 1 (11) | 15.7 | 9 | 1 (11) | 12.2 | 18 | 2 (11) | 14.0 |
| *Rv1979c*(50) | *Rv1979c* | 10 | 2 (20) | 13.1 | 11 | 0 (0) | 0.0 | 21 | 2 (10) | 13.1 |
| *Rv1997*(50) | *ctpF* | 11 | 1 (9) | 26.7 | 9 | 1 (11) | 11.2 | 20 | 2 (10) | 19.0 |
| *Rv2711*(37) : *Rv1404*(13) | *ideR* : *Rv1404* | 11 | 1 (9) | 41.7 | 9 | 1 (11) | 24.7 | 20 | 2 (10) | 33.2 |
| *Rv0446c*(39): *Rv0288*(11) | *Rv0446c*: *esxH* | 10 | 0 (0) | 0.0 | 11 | 2 (18) | 23.5 | 21 | 2 (10) | 23.5 |
| *Rv0383c*(30) : *Rv0394c*(20) | *Rv0383c* : *Rv0394c* | 11 | 1 (9) | 114.2 | 11 | 0 (0) | 0.0 | 22 | 1 (5) | 114.2 |
| *Rv1184c*(20) | *Rv1184c* | 11 | 1 (9) | 50.7 | 11 | 0 (0) | 0.0 | 22 | 1 (5) | 50.7 |
| *Rv2542*(39): *Rv2393*(11) | *Rv2542*: *Rv2393* | 10 | 0 (0) | 0.0 | 12 | 1 (8) | 22.0 | 22 | 1 (5) | 22.0 |
| *Rv0594*(50) | *mce2F* | 10 | 0 (0) | 0.0 | 12 | 1 (8) | 12.0 | 22 | 1 (5) | 12.0 |
| *Rv0594*(45): *Rv1966*(5) | *mce2F*: *mce3A* | 10 | 0 (0) | 0.0 | 12 | 1 (8) | 11.0 | 22 | 1 (5) | 11.0 |
| *Rv0284*(50) | *Rv0284* | 10 | 0 (0) | 0.0 | 11 | 1 (9) | 30.0 | 21 | 1 (5) | 30.0 |
| *Rv1984c*(30): *Rv1986*(20) | *cfp21*: *Rv1986* | 10 | 0 (0) | 0.0 | 12 | 1 (8) | 13.0 | 22 | 1 (5) | 13.0 |
| *Rv3558*(44) : *Rv3539*(6) | *PPE64* : *PPE63* | 10 | 0 (0) | 0.0 | 11 | 0 (0) | 0.0 | 21 | 0 (0) | 0.0 |
| *Rv1980c*(28) : *Rv1984c*(22) | *mpt64* : *cfp21* | 10 | 0 (0) | 0.0 | 11 | 0 (0) | 0.0 | 21 | 0 (0) | 0.0 |
| *Rv3347c*(50) | *PPE55* | 10 | 0 (0) | 0.0 | 11 | 0 (0) | 0.0 | 21 | 0 (0) | 0.0 |
| *Rv0151c*(50) | *PE1* | 10 | 0 (0) | 0.0 | 11 | 0 (0) | 0.0 | 21 | 0 (0) | 0.0 |

^1^The grey shading denotes peptide pools that were chosen for clinical validation.

^2^ The EsxJ peptide pool contains peptides representing 5 homologous genes that differ at only up to three amino acids. Rv1038c, Rv1197, Rv2347c, Rv3620c, Rv1792.

^3^These two peptide pools represent distinct regions of this large gene.

^4^The Mtb9.9 peptide pool contains peptides representing 5 homologous genes that differ at only up to nine amino acids. Rv1037c, Rv1198, Rv2346c, Rv3619c, Rv1793
