# Supplementary material for: Comprehensive definition of human immunodominant CD8 antigens in tuberculosis
Source: NPJ Vaccines. 2017 Apr 3;2:8. doi: 10.1038/s41541-017-0008-6 (PMC5538316; doi:10.1038/s41541-017-0008-6)
Supplement: Supplementary file 9 — Supplementary Information [file 41541_2017_8_MOESM9_ESM.docx]

# Supporting Information Summaries (All files in Word)

### Fig S1. Composition of Peptide Pools Comprising Peptide Library

### (A) The percentages of peptide pools that represent various numbers of proteins (in part or the entirety) are depicted. Of those peptide pools representing 3 or more proteins, 39 (5%) represented three and 1 (<1 %) represented 4 proteins.

### (B) The percentages of peptide pools that required various numbers of peptide pools to represent each protein are depicted. Six peptide pools (n = 8), seven peptide pools (n = 2), eight peptide pools (n = 4), and nine peptide pools (n = 2) were required to represent large proteins and one each, of six very large proteins, was represented by 10, 12, 14, 17, 18, or 19 peptide pools.

### Table S1. Subject Enrollment for Peptide Library Screens. Table S1 list targets goals and numbers of individuals screened, apheresed, and for whom interpretable library screens were obtained for each group.

### Table S2. Immunodominant Peptide Pools: Table S1 lists 49 peptide pools representing part or all of 74 unique proteins, which meet the definition of immunodominant antigens (top 5% of positive responses in three or more of the twenty subjects tested).

**Table S3. Data used to calculate proportions for pie charts displayed in Figure 2.** Table S3 contains the data used to calculate the proportions for the pie charts displayed in Figure 2.

**Table S4. Data used to calculate proportions for pie charts displayed in Figure 3.** Table S4 contains the data used to calculate the proportions for the pie charts displayed in Figure 3.

### Table S5. Experimental Evidence for Secretion: Table S2 lists 856 Mtb proteins for which there is evidence of secretion in the published literature.

### Table S6. “No Response” Peptide Pools: Table S3 lists 54 peptide pools representing part of all of 74 unique proteins, which meet the definition of “no response” (peptide pools that were not recognized by CD8^+^ T cells from any of the twenty individuals).

### Table S7. Clinical Validation of Immunodominant Peptide Pools in Mtb-Infected Individuals in Kampala, Uganda. Table S4 is a data summary of all the results of clinical validation of the immunodominant peptide pools (n = 49) in Mtb-infected Ugandan adults.
