# Supplementary material for: Comprehensive definition of human immunodominant CD8 antigens in tuberculosis
Source: NPJ Vaccines. 2017 Apr 3;2:8. doi: 10.1038/s41541-017-0008-6 (PMC5538316; doi:10.1038/s41541-017-0008-6)
Supplement: Supplementary file 10 — Supplementary Methods [file 41541_2017_8_MOESM10_ESM.docx]

**Supplemental methods: Study participant enrollment for peptide library screens**

For ex vivo CD8^+^ T cell screens of the peptide library, individuals 18 - 65 years old with a history of a positive TST and/or history of TB disease were self-referred, informed consent was obtained, and whole blood (48 ml) was drawn via venipuncture. The blood was used for HIV, HBV and HCV serologies performed by a commercial laboratory and for isolation of PBMC. PBMC were screened for levels of background in an IFN-γ ELISPOT assay. For healthy individuals with history of positive TST, PBMC were also used to assess for Mtb-specific T cell responses to distinguish true Mtb infection from exposure to BCG or atypical mycobacteria. Specifically, autologous DC were prepared from PBMC as described below. Five days later, the remaining PBMC were separated into CD4^+^ and CD8^+^ T cells using magnetic bead purification (Miltenyi Biotec, Auburn, CA, USA) and tested (250,000 cells/well) against autologous DC (20,000 cells/well) pulsed with media, or Esat6 or CFP10 peptide pools. A successful screen was defined as negative testing for infection with HIV, HBV, and HCV and, as a screen for background, CD8^+^ T cells with media alone demonstrated < 50 SFU in the IFN-γ ELISPOT assay. Finally, for healthy individuals with a positive TST, detection of CD4^+^ T cells to either CFP10 or Esat6 peptide pools was used to define LTBI. Individuals with a successful screen underwent leukapheresis to obtain PBMC to perform the library peptide screen. As individuals with active TB had to be AFB smear and culture negative prior to leukapheresis, active TB subjects underwent leukapheresis 3 – 18 months following initiation of TB therapy.
